# Estimating genealogies from linked marker data: a Bayesian approach

**DOI:** 10.1186/1471-2105-8-411

**Published:** 2007-10-25

**Authors:** Dario Gasbarra, Matti Pirinen, Mikko J Sillanpää, Elja Arjas

**Affiliations:** 1 Department of Mathematics and Statistics, University of Helsinki, Finland; 2 National Public Health Institute (KTL), Helsinki, Finland

## Abstract

**Background:**

Answers to several fundamental questions in statistical genetics would ideally require knowledge of the ancestral pedigree and of the gene flow therein. A few examples of such questions are haplotype estimation, relatedness and relationship estimation, gene mapping by combining pedigree and linkage disequilibrium information, and estimation of population structure.

**Results:**

We present a probabilistic method for genealogy reconstruction. Starting with a group of genotyped individuals from some population isolate, we explore the state space of their possible ancestral histories under our Bayesian model by using Markov chain Monte Carlo (MCMC) sampling techniques. The main contribution of our work is the development of sampling algorithms in the resulting vast state space with highly dependent variables. The main drawback is the computational complexity that limits the time horizon within which explicit reconstructions can be carried out in practice.

**Conclusion:**

The estimates for IBD (identity-by-descent) and haplotype distributions are tested in several settings using simulated data. The results appear to be promising for a further development of the method.

## Background

There are several fundamental questions in statistical genetics for which the answer would ideally require knowledge of the ancestral pedigree and of the gene flow therein. In practice, however, one will usually have available only partial information from the pedigree, and hardly any information on the accompanying ancestral allelic histories. Examples of intrinsic questions of this kind are: haplotype estimation from pedigree data [1] or from general population samples [2], pairwise estimation of the degree of relatedness between individuals in natural populations [3] or for forensic purposes [4], study of allele-sharing among affected individuals [5], generation of simulated data in a way which is compatible with observed marker genotypes (genotypic elimination; see [6,7]), estimation of population structure using multilocus genotype data [8-10], estimation of the number of founder chromosomes for given loci [11], gene mapping by combining pedigree and linkage disequilibrium information [12,13], and tracing genotyping errors in pedigrees [14].

Due to the shared generating process of inheritance pattern on the pedigree, it is not surprising that the current methods for addressing the above questions also have some similarities. One of the similarities is that several methods first attempt to generate an identity-by-descent (IBD) distribution between the individuals in the study sample, although the particular solutions for carrying this out then differ significantly from each other. There are also papers focusing only on the estimation of the IBD-distribution based on known pedigree information [1,15-17], known haplotypes [13] or known population history [18]. For population based data, there are some approaches that approximate and model the genealogical history of a sample of chromosomes from a population by using ideas from the coalescent theory [19] and its extension incorporating recombinations, expressed in terms of an ancestral recombination graph [20,21]. Applications of these ideas include haplotyping [22] gene mapping [23-25], estimating population parameters [26], and recombination rates [27]. However, in spite of the progress (see also [28,29]), the development of effective MCMC sampling methods for ancestral recombination graphs in general has been relatively slow.

This paper extends the genealogy estimation method of Gasbarra et al. [10] to the case of linked markers. Our starting point is a sample of individuals from a natural population, each being genotyped at certain marker loci, but without any direct information on their pedigree or interrelations. Our target is to provide an explicit reconstruction, in terms of a probability model, of the recent history of the genealogy connecting the sampled individuals, conditionally on the observed genotype data and available information on the demography of the population. The model is specified by the following parameters: (1) time in generations since the founding of the population, (2) the marker allele frequencies in the founder population, (3) two mating parameters α to the case of linked markers. Our starting point is a sample of individuals from a natural population, each being genotyped at certain marker loci, but without any direct information on their pedigree or interrelations. Our target is to provide an explicit reconstruction, in terms of a probability model, of the recent history of the genealogy connecting the sampled individuals, conditionally on the observed genotype data and available information on the demography of the population. The model is specified by the following parameters: (1) time in generations since the founding of the population, (2) the marker allele frequencies in the founder population, (3) two mating parameters *a *and *β *controlling the mating behaviour [30], (4) the number of males and females in each generation, and (5) the genetic distances on the marker map. Combined with an algorithm for drawing Monte Carlo samples from the conditional distribution of genealogies, this modelling framework can be applied to address, within limits of computation, all the "intrinsic questions" mentioned above.

## Methods

### Prior distribution on the configuration space

The configuration space of possible ancestral histories (Ω) has three components: the ancestral graph (or pedigree) specifying the relationships between individuals, the paths of alleles of these individuals at the marker loci, and the types of the founder alleles introduced into the ancestral graph via the founder individuals. The probability model on the configuration space is similar to the one described by Gasbarra et al. [10] except that in this study the linkage between marker loci is allowed. Due to the similarities we give here only a brief summary of the model.

#### Ancestral graph

For pedigrees we use the probability model introduced by Gasbarra et al. [30]. The model considers an isolated population with non-overlapping generations indexed backwards in time by *t *= 0, 1, ..., *T *with *t *= 0 referring to the present and *t *= *T *to the founder generation. The population is characterized by four sets of parameters: ${N^{\prime }}_{t}$, ${N^{\prime \prime }}_{t}$, *α*_*t *_and *β*_*t*_, for *t *= 1, ..., *T*. The parameters ${N^{\prime }}_{t}$ and ${N^{\prime \prime }}_{t}$ describe respectively the number of males and females belonging to generation *t *of the population. Parameter *α*_*t *_controls the differences of reproductive success between males in generation *t*: large values of *α*_*t *_imply nearly equal numbers of children for each male whereas with small values of *α*_*t *_there will be a few dominant males who are mainly responsible for the reproduction. Parameter *β*_*t *_tunes the degree of monogamy (of males) in generation *t*: large values of *β*_*t *_lead to random mating and small values of *β*_*t *_introduce more permanent family structures into the pedigree. Naturally the roles of males and females can be changed in the model. We denote this probability measure on pedigree graphs by $P_{G}$(·).

#### Flow of alleles through the ancestral graph

We assume a fixed marker map with *L *loci, and denote the recombination fractions between loci by *ρ *= (*ρ*(*l*, *l'*) : 1 ≤ *l *<*l' *≤ *L*). Note that several chromosomes can be modelled simultaneously using the recombination fraction *p*(*l*, *l'*) = $\frac{1}{2}$ to indicate that markers *l *and *l' *lie in different linkage groups.

By definition, the genome of each individual in the ancestral graph consists of a pair of paternal and maternal haplotypes. The flow of alleles through the pedigree is determined by the grandparental origins which for haplotype *i *are denoted by *ψ*_*i *_= (*ψ*_*i*_(1), ..., *ψ*_*i*_(*L*)) ∈ {0, 1}^*L*^. The convention used here is that *ψ*_*i*_(*l*) = 0 if the allele at locus *l *of haplotype *i *is of grandmaternal origin, and *ψ*_*i*_(*l*) = 1 in the case of grandpaternal origin.

If an allele carried by an individual in generation *t *> 0 is transmitted to some individual in the present generation, we say that the allele is *ancestral*, and otherwise that it is *censored*. Since we are actually interested only in the paths of the ancestral alleles we set *ψ*_*i*_(*l*) = ∅ if the allele at locus *l *of haplotype *i *is censored.

The probability of a set $\Psi ={\left(\psi_{i}\right)}_{i\in N}$ of grandparental origins of nonfounder haplotypes on the pedigree is given by

$$P_{\psi }(\Psi )=\prod_{i\in N}\prod_{l\in \Lambda_{i}}\left[\rho {\left(j(\psi_{i},l),l\right)}^{\Delta_{i}(l)}{\left(1-\rho (j(\psi_{i},l),l)\right)}^{\left(1-\Delta_{i}(l)\right)}\right],$$

where Λ_*i *_= {*l *: *ψ*_*i*_(*l*) ≠ ∅}, *j*(*ψ*_*i*_, *l*) denotes the last uncensored locus of haplotype *i *before *l*, with the convention that *j*(*ψ*_*i*_, *l*) = -∞, if *l *is the first uncensored locus of the haplotype *i*, *ρ*(-∞, *l*) = $\frac{1}{2}$ and Δ_*i*_(*l*) = |*ψ*_*i*_(*l*) - *ψ*_*i*_(*j*(*ψ*_*i*_, *l*))| with the convention that *ψ*_*i*_(-∞) = 0.

#### Types of founder alleles

Denote by *g*_*k *_= (*g*_*k*_(*l*) : *l *= 1, ..., *L*) the unordered genotype of individual *k *and let *A *= (*g*_*k *_: *k *∈ ℱ) be the vector of founders' genotypes. Assuming linkage equilibrium at the founder generation, the probability of the founder alleles is given by

$$P_{A}(A)=\prod_{k\in ℱ}\prod_{l=1}^{L}fr(g_{k}(l);l),$$

where the population genotype frequencies *fr*(·; *l*) at each marker locus *l *are assumed given. (If Hardy-Weinberg equilibrium is assumed, we can use the population allele frequencies instead.) The genotype frequencies are extended to partially or totally censored genotypes in the obvious way. Note that the ordered founder alleles together with the grandparental origins of the nonfounder haplotypes determine the flow of alleles in the pedigree.

#### Prior distribution

Given the pedigree parameters (${N^{\prime }}_{t}$, ${N^{\prime \prime }}_{t}$, *β*_*t*_, *α*_*t*_, *T*), the population genotype frequencies and the recombination fractions between the marker loci, a configuration *ω *consisting of a pedigree *G *and a geneflow with founder alleles *A *and grandparental origins Ψ, is assigned (prior) probability

$$\pi (\omega )=P_{G}(G)\times P_{A}(A)\times P_{\psi }(\Psi ).$$

### Data and posterior distribution

Suppose that we observe the genotype data *D *= (*g*_*k*_(*l*) : *l *≤ *L*, *k *≤ *n*(0)) of *n*(0) individuals in the current generation. The posterior probability of configuration *ω *is simply

$$\pi (\omega |D)=\frac{1(\omega \in C)\pi (\omega )}{\pi (C)},$$

where C ⊆ Ω is the set of configurations that are compatible with the observed genotype data.

As it seems impossible to sample independent realizations from the posterior (see Appendix A.1) we shall use a Markov chain Monte Carlo method to perform the computations.

### Markov chain Monte Carlo algorithm

The general idea of MCMC methods and the details of our algorithm are given in the appendices, whence here we only sketch the main ideas of devising an efficient proposal distribution for a Metropolis-Hastings algorithm.

In a typical proposal move of our algorithm, a group of children in the ancestral graph try to change one or both of their parents to other possible parents of the population. This is done either by selecting the children uniformly at random from the ancestral graph, or by considering all children of a randomly chosen parent. It is necessary that the proposed new paths for the ancestral alleles carried by the children are compatible with the genes carried by the new ancestors. In order to obtain such a compatible configuration with a reasonable probability, our proposal distribution given in (8) for choosing the new parents takes into account sequentially the children's genotypes and the transmission probabilities of the alleles of prospective candidate parents at all marker loci. After choosing the new parents, we use the transmission probabilities of the new ancestors to resample the paths of the alleles carried by the children (10). Finally the new configuration is accepted or rejected according to the Metropolis-Hastings rule.

In the simplified setting of our earlier work [10] the transmission probabilities were calculated by assuming free recombinations between marker loci. When the markers are linked, the situation becomes much more difficult. Although the proposal based on independent transmission probabilities produces configurations compatible with the genetic data with high probability, a drawback is that typically these configurations contain unrealistically many recombinations. In the case of several tightly linked markers this leads to low recombination likelihood scores, and consequently low acceptance rates and poor mixing of the Markov chain. In order to avoid that, we have to include at least partially the recombination likelihood into the proposal distribution for the allelic paths. This is computationally demanding but possible through the Viterbi-Baum algorithm for hidden Markov models. In Appendix B we describe how to sample the allelic phases of an individual, jointly at all marker loci, by taking into account his/her genotypes, his/her parents' transmission probabilities, and the likelihood of the recombination pattern on the haplotypes of his/her children. This step is used sequentially to generate the new allelic paths.

Using the algorithm of Kruglyak and Lander [31], we also construct a joint sampling distribution for the allelic phases of a group of siblings and of their parents at all marker loci, combining the parental transmission probabilities with the recombination likelihood on the haplotypes of the children (see Appendix A.7.2).

The computational complexity of these sampling steps grows linearly in the number of markers, suggesting that it is not an unrealistic task to handle hundreds of linked markers.

## Results

The performance of the method was tested on two simulated data sets. The first data set was designed specifically to give information on the method's performance in the problems of haplotyping and relatedness estimation. For the former, comparisons were done with corresponding results obtained with PHASE [32] and for the latter with three existing moment estimators [33-36]. The second data set was generated using concepts from gene mapping. The purpose was to evaluate the potential advantages of using the IBD-information produced by our method over simple IBS-sharing statistics.

### Haplotyping and relatedness estimation

#### Simulated data

**Figure 1:**
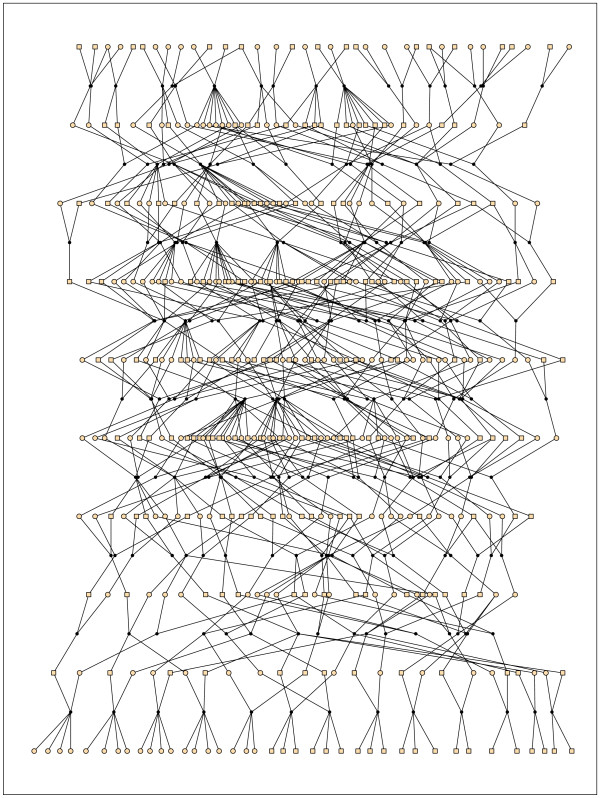
**Pedigree of the first example**. 439 individuals and 10 generations of which the youngest one consisted of the children of 13 nuclear families. Squares denote males, circles denote females. Reprinted from [10].

We considered a simulated pedigree that extended for 10 generations and contained 439 individuals (Figure 1). This pedigree was also used by Gasbarra et al. [10], as their Example III, and the details of the simulation procedure are given there.

The gene flow on the pedigree was simulated at 20 linked marker loci. All markers were polymorphic with 10 equally frequent alleles at the population level and the neighbouring loci were separated by the recombination fraction of 0.05. The simulation of genetic data was accomplished by sampling the founder alleles from the population allele frequencies and dropping them down through the pedigree in accordance with the recombination model.

#### Reconstruction

Our task was to provide possible reconstructions of the simulated pedigree and corresponding gene flow using only the unordered genotype data on the youngest generation but having no information on the underlying pedigree structure. The marker map, the population allele frequencies and the size of the population were assumed known in the reconstruction i.e. they were given the same values as in the data simulation. The mating parameters *α *and *β *can be estimated by a maximum-likelihood estimator [30] from observed family structures if the size of the base population is known. We used this approach to estimate *β *on the basis of the family structures between generations 1 and 2 in the simulated pedigree (here 0 refers to the youngest generation), resulting in the value *β *= 4 × 10^-4^. For *α *we used generation dependent values *α*_*t *_= *β*${N^{\prime \prime }}_{t}$, where ${N^{\prime \prime }}_{t}$ was the number of females belonging to generation *t *= 1, ..., 9. We ran five independent sample chains, each with a different seed for the random number generator. One of the chains was extended to 1, 000, 000 iterations whereas the other four were stopped after 500, 000 iterations. The longer run took about 10 days on a Pentium-4 2.8 GHz processor. The results for haplotyping and IBD-analyses were saved from every tenth iteration. The monitored statistics behaved very similarly across the different runs suggesting that with these data the method performs consistently regardless of the initial state. To compare the performance of the present model with our earlier model that assumed unlinked markers [10] the corresponding runs were also conducted with the simpler model.

#### Haplotyping

If there are *h *≥ 1 heterozygous loci in the multilocus genotype, then there are 2^*h*-1 ^different ways to do the haplotype assignment. Note that here we do not distinguish between the parental (paternal/maternal) origins of the haplotypes, which would further increase the number of different assignments to 2^*h*^. There is only a single correct haplotype configuration, and to measure how much our estimates deviate from it we use the concept of switch distance [2]. We say that two adjacent heterozygous loci are correctly (incorrectly) phased if the corresponding two locus haplotypes are correct (incorrect). The switch distance of the haplotype assignment is defined as the number of incorrectly phased adjacent heterozygous loci. The maximum switch distance of a haplotype configuration is one less than the number of heterozygous loci, and it is zero only for the correct configuration. For example, if the true haplotypes are (111111, 222222) then the switch distance of pair (112222, 221111) is 1 and that of pair (121212, 212121) is 5.

**Figure 2:**
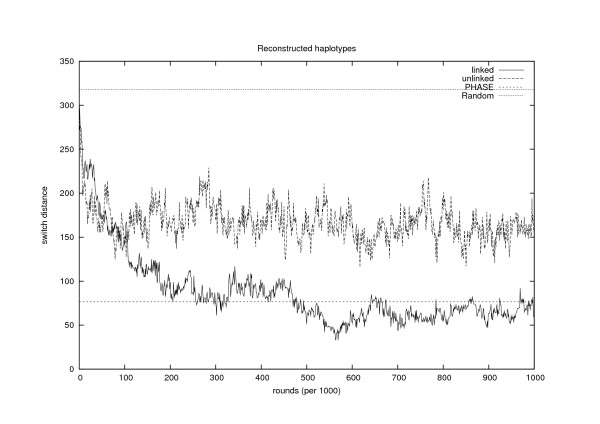
**Haplotyping**. The development of the sum of switch distances of the haplotype pairs of the youngest generation over 1,000,000 iterations, both with and without using the recombination model. The line at 318 is the expected value under random haplotype assignment and the line at 77 is the value obtained with PHASE (v.2.1).

In Figure 2 a path of the sum of switch distances of haplotypes belonging to the current generation is shown. If alleles (at heterozygous loci) were assigned to the two haplotypes randomly, then the switch distance of the haplotype pair of individual *i *would be distributed according to Binomial(*h*_*i *_- 1, $\frac{1}{2}$), where *h*_*i *_> 0 is the number of *i*'s heterozygous marker loci. The sum of switch distances would then be distributed as Binomial(*h *- *n*, $\frac{1}{2}$), where $h=\sum_{i=1}^{n}h_{i}$ and *n *is the number of individuals in the sample. In our simulated data, *n *= 39 and *h *= 675; thus, under the null model of random haplotype assignment, the expected sum of switch distances would be 318 and the corresponding standard deviation 12.6. In our five test runs the average initial value of the sum of switch distances was 295 from where it decreased during the iterations to an average value (calculated over the iterations 250,000, ..., 500,000 of the five runs) of 82.8. The corresponding average value over the iterations 500,000, ..., 1,000,000 of the longer run was 61.9. It can also be seen from Figure 2 that our recombination model significantly enhances the results from the ones attained by assuming free recombinations.

There are two kinds of haplotyping software currently available, namely pedigree-based and population-based methods. In actual fact, our data contain some close relatives, but since no relationship information is assumed to be available we are working with a general population sample. For a comparison we analysed the data with the software PHASE (v.2.1) [32]. Usually PHASE and other population-based haplotyping methods are applied to much denser marker maps where recombinations (per meiosis) are rare and the haplotypes are very likely to be inherited as single units through tens of generations. This is not the case here, but since the current version of PHASE is widely used and takes into account also recombinations, we chose it as the reference method among the existing algorithms.

We considered three different chain lengths for PHASE (100 (default), 500, and 1000 iterations) and then ran ten independent test runs for each with different choices of the seed of the random number generator. The burn-in part and the level of thinning were kept at their default values of 100 and 1 iterations, respectively.

PHASE can be requested to give the posterior probability distribution of all possible haplotypes for each individual. We measured the accuracy of these estimates by calculating the expected switch distance for each individual with respect to his/her posterior distribution of the haplotype configurations. The sums of the switch distances over all 39 individuals, averaged over ten independent runs, were 80.1, 76.6 and 76.7 for the chains of length 100, 500 and 1000 iterations, respectively. Since increasing the number of iterations from 500 to 1000 did not seem to enhance the results, we did not try to run PHASE longer. The best value PHASE gave (76.6) is shown with a dotted line in Figure 2. It seems that, at least on these data, PHASE and our algorithm have quite comparable accuracy in estimating the haplotype configurations, even though the underlying models are very different.

A clear advantage of PHASE over our method is in its speed: a single run takes only a couple of minutes whereas our algorithm was run for several days. One of the reasons is that we are modelling explicitly the whole genealogy, not just the haplotypes, and are thus able to address some other questions with the same effort. In the future we could also try using PHASE to give an initial haplotype configuration for our method.

#### Relatedness estimation

Here we consider relatedness estimation in a similar way as Gasbarra et al. [10]. Two alleles are said to be identical-by-descent (IBD) if they descend from the same ancestral allele within the pedigree. Note that two alleles may be identical-by-state (IBS), i.e. represent the same allelic form, without being IBD if two or more founder alleles happen to be of the same type. The concept of IBD thus indicates whether two contemporary alleles descended from a common ancestor that had existed since the founder generation, but it does not estimate their possible coalescent times more accurately. However, if needed, we could also capture the exact time (in generations) of these coalescing events.

In order to quantify the relatedness we denote by *r*_*ij*_(*l*) the probability that a randomly chosen allele from locus *l *of individual *i *has an IBD-copy in individual *j*. For individuals *i *and *j *we define the locus-specific relatedness coefficients *R*_*ij*_(*l*) = $\frac{1}{2}$(*r*_*ij*_(*l*) + *r*_*ji*_(*l*)) and the genome-level relatedness coefficient $R_{ij}=\frac{1}{L}\sum_{l=1}^{L}R_{ij}(l)$. Note that in the presence of inbreeding we can have *r*_*ij*_(*l*) ≠ *r*_*ji*_(*l*). However, always *R*_*ij*_(*l*) = *R*_*ji*_(*l*) and *R*_*ij *_= *R*_*ji*_.

As our input data contain no pedigree information, it seems that other methods available for IBD-estimation from such data are based on different formulas that combine the IBS-status of the markers and the known population allele frequencies to an estimate of the IBD-probability (usually *R*_*ij*_). We have compared the estimates given by our algorithm with three such moment estimators described by Lynch [33] and Li et al. [34] (LL), Lynch and Ritland [35] (LR) and Wang [36] (W).

These three methods assume unlinked loci and then combine the locus-specific results according to some weighting schemes in order to obtain estimates of the genome-level relatedness coefficients. The derivations of both LR and W are also based on the assumption of no inbreeding. Since our data violate these assumptions some additional error may be caused to the moment estimators. Moreover, it is questionable whether these moment estimators actually answer the exact question of IBD-sharing when restricted to the latest ten generations (see also [37]) as their estimates are relative to the base population defined by the allele frequencies and no exact reference point of IBD-sharing can be specified (like the founder generation in our example). On the other hand, polymorphic data sets like the one used here are advantageous for the moment estimators since in these cases IBS-sharing gives already a fairly good approximation of the actual IBD-sharing.

**Figure 3:**
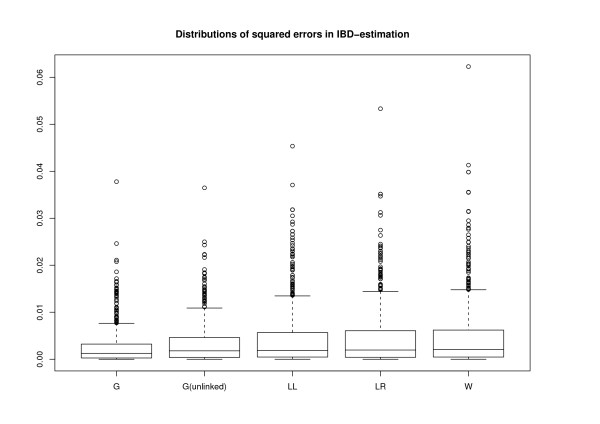
**Squared errors of relatedness estimates**. Boxplots show squared errors of all 741 pairwise relatedness coefficients *R*_*ij*_, where *i *and *j *are different individuals from generation 0. The boxes indicate the quartiles (1st, 2nd and 3rd) and the 'whiskers' cover the errors whose distance from the box is less than 1.5 times the box size. The outliers are indicated with single points. Methods used: ours (G), ours without linkage model (G(unlinked)), Lynch and Li's (LL), Lynch and Ritland's (LR) and Wang's (W).

The accuracy of the relatedness estimates was measured by squared error. Namely, we computed the true values of the coefficients *R*_*ij *_for each pair of individuals from the original genealogy and compared then the distribution of quantities (*R*_*ij *_- ${\hat{R}}_{ij}$)^2 ^between our method and the three above mentioned moment estimators. We also included results obtained with our method without modelling the linkage in order to illustrate again that the linkage model enhances the results. The distributions of the errors are shown with boxplots in Figure 3, where the letter G refers to our method. The sums of squared errors over all 741 pairs of individuals were 1.89, 2.43, 3.25, 3.27 and 3.51 for G, G(unlinked), LL, LR and W, respectively. The results for our method were calculated as the average values over the five runs of length 500,000 iterations (burn-in parts were 250,000 iterations).

**Figure 4:**
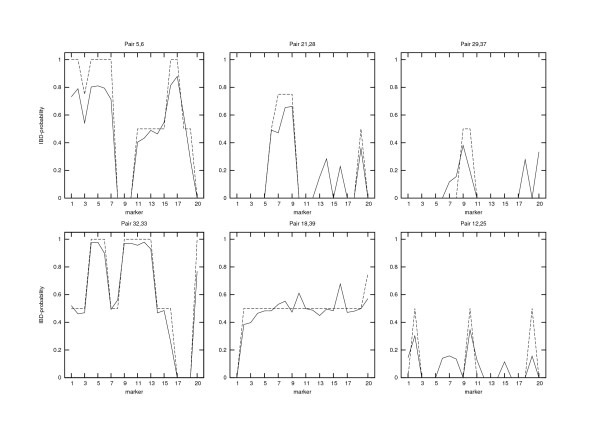
**IBD-sharing probabilities *R*_*ij*_(*l*) for six pairs of individuals from generation 0**. These individuals can be found from Figure 1 where the indexes increase from right to left (from 1 to 39). The two leftmost panels illustrate the IBD-sharing profiles of full-siblings, the upper panel in the middle is of a pair of first cousins, and the lower one describes half cousins. The two rightmost panels show the IBD-sharing between the most distant relatives that can be found in the data. The dotted lines are the exact values and the solid lines our estimates.

In gene mapping it is of interest to know the exact locations in the genome in which some group of individuals share alleles IBD (see the next example). In Figure 4 we have chosen six pairs of individuals belonging to the current generation and illustrated both the true IBD-sharing fractions *R*_*ij*_(*l*) (dotted lines) and the estimated IBD-probabilities ${\hat{R}}_{ij}$(*l*) (solid lines). It seems that our results do not significantly underestimate the true IBD-profiles. As a very large pedigree would generally result in too low IBD-estimates we may conclude that the reconstruction algorithm has not introduced many extra parents to the pedigree. In those parts of the chromosomes where our estimates exceed the true values, there is a certain amount of additional IBS-sharing for which our algorithm has not been able to completely rule out the possibility of it actually being IBD. Note that the lack of IBS-sharing already implies that there can be no IBD-sharing.

### Gene mapping

In this example we applied concepts from gene mapping to further monitor the accuracy of our reconstruction algorithm. We simulated a monogenic trait with a dominant mode of inheritance, and then investigated whether we can trace the position of a trait locus, relative to a set of marker loci, by considering suitable allele or haplotype sharing statistics. The purpose of the example is to compare the estimated IBD and haplotyping results to the simulated "true" ones and to the plain IBS data, whereas a proper extension of the model to gene mapping will be considered elsewhere (see also Discussion).

#### Simulated data

We considered a population that has grown exponentially by a factor of 1.2 during the 9 most recent generations. The founder level (assumed to be in Hardy-Weinberg and linkage equilibrium) was taken to be the 19th generation (backwards in time). The population was postulated to have maintained a constant size of 200 individuals between the 9th and the 19th generations. The method of Gasbarra et al. [30] was used to simulate a 20-generation pedigree from this population, with 400 individuals at the current (0th) generation. The parameter *α *was set to 10.0 in order to decrease the relatedness of the individuals belonging to the current generation. The monogamy parameter *β *was set to 0.001. As a result the pedigree contained 4815 individuals of whom 120 were founders.

A gene flow was simulated on the marker map containing 14 microsatellite markers separated by the recombination fraction of 0.10, and 26 SNP markers located in such a way that between any two adjacent microsatellites there were two SNPs evenly spaced with respect to genetic distance. The allele frequencies were sampled from the Dirichlet distribution with all parameters equal to 1. For microsatellites there were 10 different alleles and the SNPs were biallelic at the founder level.

Having simulated the pedigree and the gene flow we fixed an additional trait locus half way between (SNP) markers 20 and 21 and simulated the segregation of the founder alleles at that locus in accordance with the inheritance patterns of the flanking markers and the Haldane recombination model. We chose one particular founder allele at the trait locus and collected all of its 44 carriers from the current generation to form our sample.

#### Reconstruction

The question was whether we could spot the trait locus by comparing the values of an IBD-sharing statistic of the sampled individuals in different marker loci. The idea is that the carriers should share more alleles IBD near the trait locus than elsewhere on the chromosome. For computational reasons, and also in order to violate the assumption of the founder generation being in exact Hardy-Weinberg and linkage equilibria, the time horizon was in the reconstruction set to 9 generations instead of 19 that was used in the simulation. Considering the history only 9 generations backwards is likely to produce challenges for the reconstruction, since in the simulated data there were altogether six different copies of the trait allele at the 9th generation, all of whom had descendants among our sample. On the other hand, when extending the analysis for tens of generations backwards in time the exact number of generations being considered is likely to become less important, and also it is less likely that we could find out the exact generation in which each coalescing event actually occurred.

The population allelic frequencies and the population size were considered known. For *β *the previously estimated value 4 × 10^-4 ^was used and again *α*_*t *_= *β*${N^{\prime \prime }}_{t}$ where ${N^{\prime \prime }}_{t}$ was the number of females belonging to generation *t*, *t *= 1, ..., 9 (the current generation has index 0). The algorithm was run for 100, 000 iterations, which took about two days on a Pentium-4 2.8 GHz processor. The results were saved from every tenth iteration and averaged over two independent runs.

#### Results

Let us denote by ℱ the set of founders of a pedigree and by S the set of individuals in our sample. For each marker *l *and each individual *i *denote by $G_{i}(l)=\{g_{i}^{\left(1\right)}(l),g_{i}^{\left(2\right)}(l)\}$ the marker genotype of *i *at locus *l *. Enumerate all 2|ℱ| founder haplotypes and let $F_{i}(l)=\{f_{i}^{\left(1\right)}(l),f_{i}^{\left(2\right)}(l)\}$ be the founder alleles of *i *at locus *l*, where $f_{i}^{\left(k\right)}$(*l*) is the label of the founder haplotype from which the allele $g_{i}^{\left(k\right)}$(*l*) originates (*k *= 1, 2). We consider the following allele sharing statistic. Let Min(S; *l*) be the size of a smallest set *V *⊆ {1, ..., 2|ℱ|} of founder alleles for which

$$f_{i}^{\left(1\right)}(l)\in V\text{ or }f_{i}^{\left(2\right)}(l)\in V,\text{ for all }i\in S.$$

**Figure 5:**
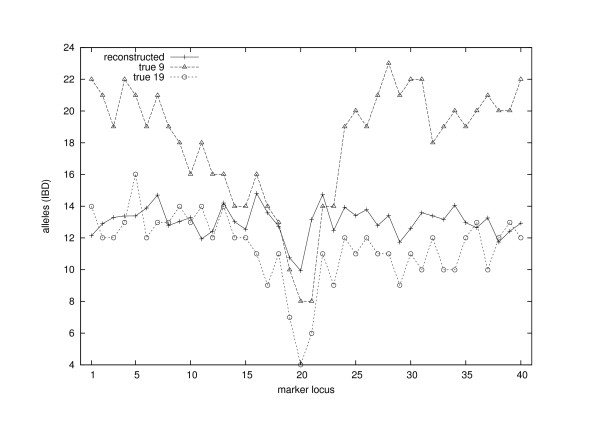
**IBD-sharing among 44 sampled individuals at each marker locus**. The statistic Min(S; *l*) was calculated from the original situation with respect to the 19th generation (original founder level) and the 9th generation and from a reconstruction over 9 generations.

Note that such a minimal *V *does not have to be unique, but our interest lies in the unique size of these sets. Finding such a minimal set *V *is an instance of a well-known NP-complete problem of finding a minimum vertex cover for a given graph. This can be seen by considering the graph where the founder alleles are vertices and for each *i *∈ S there is an undirected edge between $f_{i}^{\left(1\right)}$ and $f_{i}^{\left(2\right)}$. Graph theoretic formulation of the problem made it possible to compute the exact values of Min(S; *l*). (A brute-force search over 2^44 ^different sets at each locus and on each iteration would not be feasible.) The same statistic (with a different sign) is called $T_{\text{blocks}}^{\text{dom}}$ in [5] where it was reported to work well for extended pedigrees and dominant traits. In Figure 5 we have plotted the values of Min(S; *l*) at each marker locus, both for the reconstruction (averaged over iterations) and for the true situation with two choices for the founder generation (9th and 19th generations). All three plots have their minimum at the marker locus 20, in good agreement with the trait locus lying half way between the loci 20 and 21.

It seems that generally the number of different founder alleles in the reconstruction is more similar to the original situation in the 19th generation than to that in the 9th generation, even though the reconstruction was actually done for 9 generations. This suggests that, as we reconstruct more generations backwards in time, the assumption of the founder generation being in Hardy-Weinberg and linkage equilibria may become more important than the actual number of generations considered. In other words, the algorithm may try to squeeze the original pedigree and gene flow to the given time horizon. On the other hand, it is unlikely that the postulated genetic equilibria would hold to a very close approximation among a set of founders of a population isolate.

**Figure 6:**
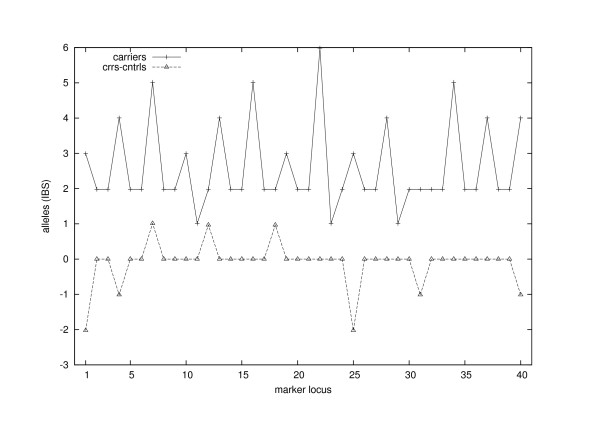
**IBS-sharing among 44 sampled individuals at each marker locus**. The upper curve illustrates similar statistic as Min(S; *l*) but calculated from IBS-status. The lower curve displays the difference Min(S; *l*) - Min(C; *l*), where C is a control group. No signal of the trait locus between the markers 20 and 21 can be found from these IBS-statistics.

No similar drop in allele numbers can be seen near the trait locus in mere IBS-sharing statistics among the sampled individuals. In Figure 6 we have plotted the IBS-based Min(S; *l*) statistics that were calculated by replacing the founder labels $f_{i}^{\left(k\right)}$(*l*) in the definition above with the corresponding allele types $g_{i}^{\left(k\right)}$(*l*). Since allele frequencies may have a strong effect on the expected number of different IBS-alleles, we have chosen a control group C of 44 individuals randomly among the 356 non-carriers belonging to the current generation of the original pedigree. The lower curve in Figure 6 illustrates the differences Min(S; *l*) - Min(C; *l*). Staying non-negative near locus 20, it does not seem to give a signal of the trait locus. We also compared the entropies of the (IBS) allele distributions of carriers and controls (results not shown) but did not find any excess allele sharing near the trait locus.

As an alternative measure of genetic similarity we can also monitor how long haplotype segments the sampled individuals share in different parts of the genome. For a given locus *l *the haplotype sharing statistic HSS(*l*) is computed as the average sharing between all distinct haplotype pairs (*h *and *k*) at that locus in the sample. We say that the sharing *s*_*hk*_(*l*) is zero if the corresponding alleles at locus *l *are inherited from different founder haplotypes, otherwise *s*_*hk*_(*l*) is the length (in genetic distance) of the corresponding overlapping IBD-segments. Following [30] the value of the haplotype sharing statistic at locus *l *in the sampled group of individuals was evaluated using the formula

$$\text{HSS}(l)=\frac{1}{n(2n-1)}\sum_{h=1}^{2n}\sum_{k<h}s_{hk}(l),$$

where *n *= |S| (see also the statistics in [38-40]).

**Figure 7:**
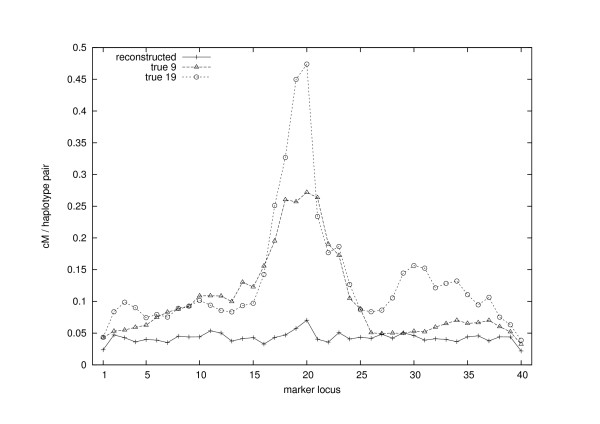
**Haplotype sharing among 44 sampled individuals at each marker locus**. HSS is calculated for the original situation with respect to two different founder levels (19th and 9th generations) and for the reconstruction (9 generations). The signal in the reconstruction is very weak compared to the true situation.

In Figure 7 we show the values of the haplotype sharing statistics for both the reconstruction and the true situation, the latter with two different choices of the founder level (the 9th and the 19th generation). The curves calculated from the true allele paths show clearly that the sampled individuals share longer haplotypes near the trait locus than elsewhere in the chromosome, especially in the direction of the 20th marker locus. These facts are also present in the reconstruction, but the signal is much weaker.

The questions that remain can now be stated as follows: (i) How much of the complete information do the mere genotype data on the youngest generation contain, and (ii) how strong a signal one can expect to get from this kind of data with any computational method? We use a long chromosomal segment (1.45 Morgans) with a quite sparse marker map (over 3 cM between adjacent loci), as we are interested in the recombination process. Difficulties with these data may arise because recombinations are frequent and mix up shorter segments in various ways. In addition, the sampled individuals are more distantly related to each other in this example than in the previous one, as can be seen from the average values of *R*_*ij *_with respect to the 9th generation, (0.05 and 0.19, respectively, in this and in the previous example).

Haplotyping is easier for our method when there are siblings or other close relatives in the sample, since their data tend to cluster to the same families also in the reconstruction and thus increase locally the amount of information on those parts of the pedigree. This can be seen, for example, in Figure 4 where the estimates of IBD-sharing are very close to the true values for siblings (the leftmost pictures) and somewhat less accurate for more distant relatives (the rightmost pictures). Some further enhancements in the estimates might also be achieved if we knew some parts of the pedigree and were able to utilize this information in our algorithm. Such possibilities will be considered in our future work.

On the other hand, we cannot completely rule out the possibility that the relatively weak signals in haplotype sharing would be influenced by the slow mixing of the MCMC sampler.

## Discussion

The main motivation leading to this study was to provide a common methodological basis for considering a number of inter-related fundamental questions in statistical genetics, by extending our earlier work [10] to linked marker data. Our approach can be seen to complement the methods that are used in the reconstruction of coalescents. In particular, both approaches start from DNA samples taken from present day individuals and then make an attempt to trace back their common genetic origins. In the original coalescent analysis the main focus is on attempts to reconstruct the underlying evolutionary history, driven by mutations [19]. To do this, one proceeds backwards along germ lines looking for Most Recent Common Ancestors (MRCA's), every time coalescing two lines when a common ancestor for them is postulated, and finally ending with the root of the resulting tree structure. Later the coalescent theory has been extended in several directions [41], most notably to ancestral recombination graphs (ARG's) [20,21] that include the recombination process. Common to the coalescent theory and its extensions is that the relevant time scale for them is usually of the order of thousands of generations or even much longer. On balance, generally one then considers only relatively short aligned sequences of DNA at a time. The present method is analogous to the search for MRCA's in that it, too, can be seen as a search for chromosomal areas shared by some individuals in the sample, and is carried out by sampling explicit hypothetical reconstructions of the past. Here, however, the genealogies are assumed to be driven by mating and meioses and the effect of mutations is ignored. Also the time scale in which these reconstructions are carried out is very much shorter, of the order of tens of generations. But then they are carried out by jointly considering marker loci that cover much wider chromosomal areas such as whole chromosomes, or even the entire genome. Note also that our model builds on an explicit consideration of diploid chromosomes, which is a source of substantial technical complications in the computations. Thus, in trying to resolve questions concerning shared ancestral origins of the marker alleles, we also allow for the possibility of inbreeding, that is, loops in the ancestral graph. The original coalescent process considers the genealogy of a random sample of genes from a very large and randomly mating population whence the sample size has a large effect on the coalescent time. In contrast, if we consider closely related or ascertained (for some phenotype) individuals in an effectively smaller population, then even a small sample is likely to find common ancestry within our framework. Thus the main factor in determining the rate of coalescences in our method is the relatedness structure that the data contain, not necessarily the number of sampled individuals in the data. Additionally, we emphasize that in our model (as in ARG's) the coalescences may involve only small parts of the haplotypes and thus the coalecences are not tied to the number of individuals in the first place. Namely, after a few generations backwards in time, the haplotypes of any sampled individual may have split into tens of parts, each having its own genealogical tree.

Reconstructing plausible pedigrees of the sampled individuals conditionally on the observed marker data always requires some knowledge about the recent history of the population. Here such information is provided in the form of postulated parameter values for controlling population growth and mating, as well as assuming that the members of the founder generation are in linkage and Hardy-Weinberg equilibrium. Unrelated founders in linkage equilibrium seems to be a common assumption in pedigree analyses. This assumption is likely to be unrealistic especially when it is applied to small pedigrees whose founders lie only a few generations backwards in time (as in traditional linkage analysis). On the other hand, extending the founder level to some tens of generations farther back in time, as is done here, allows more realistic modelling of the relatedness structure within a few of the most recent generations (i.e. within those generations that contain the sampled individuals).

When assessing the usefulness of our approach in practical applications based on real data it will be important to assess the sensitivity of the results to variations in the tuning parameters. In the gene mapping example one may also enquire how strongly the results would depend on the number of generations that are considered in the reconstruction. As noted in [10] the relation between the concept of generation in the model and its counterpart in the real population is not straightforward and varies as a function of marker data and parameter values.

Our numerical examples illustrated how one can usefully summarize the relevant posterior information contained in an MCMC sample of ancestral graphs by considering certain statistics of interest, such as those describing the relatedness between a pair of individuals at different marker loci. One can then think of the sampled pedigrees as being merely vehicles that alleles need to find their way through the pedigree from the founders to the study sample, or as nuisance parameters that will ultimately be integrated out from the results. In view of the enormous size of the sample space of ancestral graphs, it is the relative robustness of these summary statistics to the exact pedigree and gene flow information which makes our approach based on MCMC sampling at all feasible.

Our approach becomes soon computationally infeasible as the number of generations in the reconstruction increases since the task of finding suitable proposals requires computations whose complexity grows rapidly with an increasing depth of the pedigree. In our numerical examples we used a single desk computer. However, more computational power is needed to handle larger data sets and/or denser marker panels (e.g. SNP data) in a reasonable time. We have sketched a tempering version of the algorithm in which several chains run in parallel (see also [42]). The idea there is to improve the mixing of the sampler by giving each chain its own "temperature" that then controls the weight of each recombination likelihood in the acceptance ratios of the Metropolis-Hastings updates. The higher the temperature, the more easily the proposals are accepted, but the results are monitored only for the chain at the lowest temperature where the recombination likelihood is not relaxed at all. After a certain prespecified number of iterations the chains at neighbouring temperatures compare their configurations in terms of their respective likelihood values and then may swap the temperatures according to a suitable rule. The idea is that if some chain finds a good configuration, this configuration can move gradually towards the lowest temperature from which the results are then collected.

Another method, also using parallel Markov chains, is provided by the Feynman-Kac-Metropolis algorithm [43]. Given a Metropolis-Hastings algorithm with target distribution *π*(*x*), proposal distribution *q*(*x *→ *y*) and acceptance probability *a*(*x *→ *y*), one can construct a system of *N *interacting Markov chains $\left(X_{t}^{\left(1\right)},...,X_{t}^{\left(N\right)}\right)$ as follows. At time *t*, given the previous state of the system $\left(X_{t-1}^{\left(1\right)},...,X_{t-1}^{\left(N\right)}\right)$, we sample independently proposal states ${\hat{X}}_{t}^{\left(1\right)},...,{\hat{X}}_{t}^{\left(N\right)}$ from the respective proposal distributions *q*$\left(X_{t-1}^{\left(i\right)}\to {\hat{X}}_{t}^{\left(i\right)}\right)$, *i *= 1, ..., *N*. Then, for *i *= 1, ..., *N*, with probability *a*($X_{t-1}^{\left(i\right)}\to {\hat{X}}_{t}^{\left(i\right)})$ we take $X_{t}^{\left(i\right)}={\hat{X}}_{t}^{\left(i\right)}$, and otherwise sample $X_{t}^{\left(i\right)}$ from the set {${\hat{X}}_{t}^{\left(1\right)},...,{\hat{X}}_{t}^{\left(N\right)}$} by assigning the probability

$$a(X_{t-1}^{\left(j\right)}\to {\hat{X}}_{t}^{\left(j\right)})/\left(\sum_{j=1}^{N}a(X_{t-1}^{\left(j\right)}\to {\hat{X}}_{i}^{\left(j\right)})\right)$$

to the choice $X_{t}^{\left(i\right)}={\hat{X}}_{i}^{\left(j\right)}$. In this way the *N *chains interact and the particle system explores the target distribution's landscape more efficiently than a system of *N *independent Metropolis chains based on the same proposal kernel *q*(*x *→ *y*). Although the *N*-fold product distribution *π*^⊗*N *^is not the invariant distribution of the *N*-particle system, it has been shown that as *N *→ ∞ and *t *→ ∞, the empirical distribution $\left(N^{-1}\sum_{i=1}^{N}\delta_{X_{t}^{\left(i\right)}}\right)$ converges to the target distribution *π*.

We conclude by some remarks concerning gene mapping. Often data used in genetic mapping studies consist of a number of nuclear or extended families, each formed by first ascertaining an affected individual (proband) and then collecting marker and/or phenotype data on close relatives of the proband. If this is the case, it is natural to make use of the known family structure and of the marker data that may be available, for example, from the siblings and the parents of the proband. Considering the gene flow within each such small known pedigree, and making use of this information in a phenotype or penetrance model, will then correspond to "ordinary" linkage or QTL-analysis. However, particularly in data sets in which all probands are collected from a genetic isolate, as is often the case, also these small pedigrees can be assumed to share, in the sense of IBD, some part of their ancestry. In such cases, there is growing interest in the current literature in modelling the relatedness between founder individuals (e.g., [12,13,44]). In the currently existing approaches the recent shared ancestry is modelled only at the putative QTL-position or estimated separately for each marker/QTL (based on flanking marker loci), whereas here we consider this question jointly at all marker loci. Note also that, by making use of the Haldane map function, this gives us a handle for doing the same, in terms of probabilities, on the intervals between flanking markers. In a near future our plan is to modify the present reconstruction method in a way which allows us to fix the known parts of the pedigree and the corresponding marker information to the extent in which it is known, and then apply the reconstruction algorithm for building "bridges between these islands".

It seems likely that no single method can perform equally well on the whole spectrum of different types of genetic data currently available. Indeed, interplay of several methodological approaches will be crucial during the future gene mapping studies. The main role of our method may be in the initial stage of a genome-wide mapping project when interesting regions are sought using a marker spacing that is measured in centimorgans and the pedigree records are not complete. In the study of complex disease traits our method can be applied to estimate the haplotypes and/or relatedness structure which can then be used as input parameters for subsequent QTL or association mapping (e.g. [45]). In order to provide a more systematic approach to this problem, we are currently planning to build an even larger Bayesian model which would allow us to combine these two stages of analysis. This would involve expanding the present method further by adding to the model one more layer of hierarchy corresponding to the underlying genetic architecture of the trait [45-48]. In such a large integrated method, generation of IBD and haplotype distributions as well as screening of QTL-positions could all be performed as parts of a joint analysis. At least in principle, the number of contributing loci and their positions, the size of their effects, interactions within and between genes and environmental factors, as well as the mode of a gene action, could be analysed by such a method. Of course, the computational requirements for this kind of gigantic model are even larger than for the current method, and the practical implementation will be a major computational challenge.

## Conclusion

We have implemented an algorithm for analyzing recent history of linked multilocus genotype data sampled from an isolated diploid population. We model the paths of the observed alleles through tens of generations by explicitly including all ancestral individuals and corresponding meioses into the possible ancestral configurations. Thus we are extending the methods that estimate gene flows on fixed pedigrees to the case where also the pedigrees need to be estimated.

We have tested the method on the problems of haplotyping and IBD-estimation. In both cases the method performs well compared to some widely used existing methods. We have also illustrated how our estimates for IBD-sharing are more informative than a simple IBS-sharing statistic on a tentative example on gene mapping.

Our experiences reported here and in [10] encourage us to develop the method further. Indeed, the current version of the method can be seen as a general tool for estimating genealogical relationships between sample units. In more complex applications, such as gene mapping, it can serve as a basis for extended models.

## Authors' contributions

DG designed, implemented and reported the MCMC algorithm. MP tested the program, implemented the parts needed in the applications and conducted the example analyses. EA initiated and coordinated the study. All authors designed the example analyses jointly, participated in writing the manuscript and read and approved the final version of the text.

## Appendices

In the following two appendices we describe the MCMC algorithm in more detail.

Appendix A first discusses why we are not able to sample from the posterior without MCMC. Then we briefly summarize the theoretical background of the MCMC methods, describe a way to generate an initial configuration for the chain, and finally explain the block-updates that are used to propose new states for the chain.

Appendix B introduces an algorithm to sample the phases of the parent's genotype given partially observed haplotypes of his/her children. It is used in our block-updates described in Appendix A, but it may also turn out to be useful in other settings.

We use the notation introduced in the Methods section. In addition we index the individuals in the pedigree starting from the present generation and assign labels 2*k *- 1 and 2*k *respectively to the paternal and maternal haplotype of individual *k*. If the allele at locus *l *of haplotype *i *is ancestral, we denote its type by *h*_*i*_(*l*) ∈ *E*_*l*_, otherwise setting *h*_*i*_(*l*) = ∅, where *E*_*l *_is the set of alleles at locus *l*. Thus for each individual *k *and locus *l*, *g*_*k*_(*l*) = {*h*_2*k*-1_(*l*), *h*_2*k*_(*l*)}.

## A Sampling from the posterior distribution

### A.1 Possibility of sampling without Markov chain Monte Carlo

We want to study the posterior distribution (1) by using a Monte Carlo method, so we need an algorithm for sampling random realizations from the posterior. In a way, constructing such an algorithm corresponds to time-reversing a Markov process in a discrete state space, where ideally we could write down the generator of the reversed dynamics jointly for the ancestral graphs and the allelic paths, and the posterior distribution of the haplotypes in the present generation given the data, and then sample directly independent realizations of the backward process. However, the number of terms involved in such summations grows extremely rapidly with the sample size *n*(0), the time horizon *T *and the number of loci, whence the computation of the reverse generator is not feasible in the real-life data problems we have in mind. Next we briefly examine some alternative ideas.

First, we could proceed naively, by sampling repeatedly from the prior until we obtain a realization *ω *∈ C. This is problematic when *π*(C) is very small, as is the case here, and on average it takes far too many attempts to obtain even one realization from C.

Alternatively, since Ω is a finite set for any given (*n*(0), *T*, *L*), we could compute and draw samples directly from the posterior just by summing *π*(*ω*)1(*ω *∈ C) over *ω *∈ Ω. Again, this is not feasible in practice when |Ω| is very large.

As a third possibility, we could hope to have better chances for direct simulation by using hidden Markov model techniques. We discuss briefly this idea, since it is used later in the construction of the MCMC algorithm. Given the ancestral graph *G *with |*G*| individuals, we construct, according to the recombination model, a Markov chain (*ψ*(1), ..., (*ψ*(*L*)), where *ψ*(*l*) = (*ψ*_2*k*-1_, *ψ*_2*k *_: *k *≤ |*G*| - |ℱ|), ℱ is the set of founders and *ψ*_*i*_(l) denotes the grandparental origin of the allele (ancestral or censored) at locus *l *of haplotype *i*. In order to preserve the Markov property across consecutive loci, the configuration space has to contain also the grandparental origins of the censored alleles. For the individuals in the present generation (*t *= 0) we also need a random parental phase matrix (*ϕ*_*k*_(*l*) : *k *= 1, ..., *n*(0), *l *= 1, ..., *L*) with entries *ϕ*_*k*_(*l*) ∈ {0, 1} to determine the haplotypes. The random variables (*ϕ*_*k*_(*l*)) are a priori i.i.d. with *P*(*ϕ*_*k*_(*l*) = 0) = *P*(*ϕ*_*k*_(*l*) = 1) = $\frac{1}{2}$ and together the pairs (*ψ*(*l*), *ϕ*(*l*)) form a Markov chain on the finite state space {0, 1}^*d*^, where *d *= 2(|*G*| - |ℱ|) + *n*(0). At each locus *l*, the vector *Y*(*l*) = (*g*_*i*_(*l*) : *i *= 1, ..., *n*(0)) is observed. The corresponding likelihood contribution from locus *l *is given by

$$P(Y(l)|\psi (l),\phi (l),G)=1(\omega \in C)\prod_{k\in ℱ}fr(g_{k}(l);l),$$

where the genotypes of the founders are determined by the triple (*Y*(*l*), *ϕ*(*l*), *ψ*(*l*)).

By using the Viterbi algorithm, it is possible to sample directly the random vector ((*ψ*(*l*), *ϕ*(*l*)) : *l *= 1, ..., *L*) conditionally on the data *Y*. Kruglyak and Lander [31] proposed an efficient implementation of the Viterbi algorithm using Fourier transforms on the commutative group {0, 1}^*d *^(see also section A.7.2). The Viterbi algorithm could also be used to integrate out the allelic paths and to obtain the marginal likelihood *P*(*Y*(1), ..., *Y*(*L*)|*G*) of the data set *Y *given the ancestral graph *G*. We would then be left with the problem of sampling from a posterior distribution on the finite space G of ancestral graphs with *n*(0) roots spanning *T *generations backwards in time, with probabilities proportional to

*P*(*G*) × *P*(*Y*(1), ..., *Y*(*L*)|*G*).

There are two problems in this approach, however: Firstly, the Kruglyak-Lander algorithm can be implemented only for small values of *d*, say *d *≃ 20 at most. Secondly, the number of possible ancestral graphs grows extremely rapidly with *n*(0) and *T*.

In summary, the direct sampling methods described above will work only for small ancestral graphs, and next we shall describe the general ideas of Markov chain Monte Carlo methods that can yield approximate results also in more complex settings.

### A.2 Metropolis-Hastings algorithm

The Metropolis-Hastings algorithm is a recipe to construct a reversible and ergodic Markov chain (*Z*_*n *_: *n *∈ IN) with a given invariant probability distribution *μ*(*z*) on a state space Z[49]. The method can be used for general state spaces but we shall restrict our considerations to the case where Z is finite. We choose a *proposal transition kernel Q*(*z *→ $\bar{z}$) and define the corresponding *acceptance probability*

$$a(z\to \bar{z})=\min\left\{1,\frac{\mu (\bar{z})Q(\bar{z}\to z)}{Q(z\to \bar{z})\mu (z)}\right\}.$$

Note that in order to compute *a*(*z *→ $\bar{z}$) we need to know the target measure *μ*(*z*) only up to a normalizing constant. The corresponding transition kernel of the Markov chain (*Z*_*n *_: *n *∈ IN) is then given by

$$K(z\to \bar{z})=a(z\to \bar{z})Q(z\to \bar{z})+1(\bar{z}=z)\left(1-\sum_{z^{\prime }\in Z}a(z\to z^{\prime })Q(z\to z^{\prime })\right).$$

In other words, given the previous state *z*_*n*-1_, we draw a random sample $\bar{z}$ from the proposal distribution *Q*(*z*_*n*-1 _→ $\bar{z}$) and then let *Z*_*n *_= $\bar{z}$ with probability *a*(*z*_*n*-1 _→ $\bar{z}$), otherwise setting *Z*_*n *_= *z*_*n*-1_. The distribution of the initial state *Z*_0 _together with the transition kernel specifies the distribution of the Markov chain (*Z*_*n *_: *n *∈ IN). If the chain is irreducible, the construction results in an ergodic Markov chain for which the law of large numbers holds meaning that for any integrable function *f*(*z*),

$$\underset{n\to \infty }{\lim}\frac{1}{n}\sum_{i=1}^{n}f(Z_{i})=\sum_{z\in Z}f(z)\mu (z),$$

with probability one. This is a useful result when we would like to approximate numerically the expectation on the right hand side, but there is no practical algorithm producing i.i.d. realizations from *μ*(*z*). The choice of the proposal distribution *Q*(*z *→ $\bar{z}$) determines the mixing properties of the Metropolis chain (*Z*_*n*_). If mixing is too slow, the Metropolis chain is useless for Monte Carlo computations.

It is also possible to combine different Metropolis kernels *K*_*i*_(*z *→ $\bar{z}$), *i *∈ IN, with the same invariant distribution *μ *and still obtain a Markov chain (*Z*_*n*_) such that (4) holds. One way is to consider adistribution (*p*_*i*_) on the non-negative integers, and define a new transition kernel as the mixture

$$K(z\to \bar{z})=\sum_{i=0}^{\infty }p_{i}K_{i}(z\to \bar{z}).$$

Another possibility would be to combine the proposals into the new proposal

$$Q(z\to \bar{z})=\sum_{i=0}^{\infty }p_{i}(z)Q_{i}(z\to \bar{z}),$$

where the mixing distribution is allowed to depend on the current state *z*, and the corresponding transition kernel is computed by using (2) and (3).

#### A.2.1 Gibbs' updates

It is sometimes possible to represent the state space as a Cartesian product Z = $Z^{\prime }\times Z^{\prime \prime }$. For *z *= (*z'*, *z"*) ∈ $Z^{\prime }\times Z^{\prime \prime }$, the proposal kernel of a Gibbs update is given by

$$Q((z^{\prime },z^{\prime \prime })\to ({\bar{z}}^{\prime },\bar{z^{\prime \prime }}))=1({\bar{z}}^{\prime }=z^{\prime })\mu ({\bar{z}}^{\prime \prime }|Z^{\prime }=z^{\prime })$$

and we obtain another Gibbs' update by inverting the roles of *z' *and *z"*. The corresponding acceptance probability satisfies *a*((*z'*, *z"*) → (${\bar{z}}^{\prime },{\bar{z}}^{\prime \prime }$)) ≡ 1. However, it is not always the case that we can use the Gibbs update for a given decomposition of the state space, since this requires direct sampling from the conditional distribution *μ*(*z"*|*Z' *= *z'*).

### A.3 MCMC with auxiliary variables

Here we explain a procedure which is used frequently in the MCMC-literature (see Appendix 2 in [50]). Suppose we have constructed a Markov chain (*Z*_*n*_) on the (finite) state space Z with a given equilibrium distribution *π*(*z*). Consider an enlarged state space $\tilde{Z}=(Z\times Y)$, together with a stochastic kernel *p*(*y*|*z*) : Y×Z → [0, 1]. Define the probability measure $\tilde{\pi }$ (*z*, *y*) = *π*(*z*)*p*(*y*|*z*). The idea is to explore the marginal distribution $\pi (z)=\sum_{y\in Y}\tilde{\pi }(z,y)$ by constructing an ergodic Markov chain ($\tilde{Z}$_*n*_) on the enlarged state space $\tilde{Z}$ with equilibrium distribution $\tilde{\pi }$(*z*, *y*). In what follows, we assume that for every *z *∈ Z we have an algorithm to sample a random realization of *Y *from *p*(*y*|*z*) and a numerical procedure to compute this conditional distribution.

If *K*(*z *→ $\bar{z}$) is a transition kernel which is reversible with respect to *π*(*z*), we can always consider on the enlarged state space $\tilde{Z}$ the transition kernel

$$\tilde{K}((z,y)\to (\bar{z},\bar{y}))=K(z\to \bar{z})p(\bar{y}|\bar{z})$$

which will automatically be reversible w.r.t $\tilde{\pi }$(*z*, *y*). In particular, the transition kernel $K((z,y)\to (\bar{z},\bar{y}))=1(\bar{z}=z)p(\bar{y}|\bar{z})$ is reversible w.r.t $\tilde{\pi }$(*z*, *y*).

Next consider the Metropolis transition kernel with joint proposal distribution $\tilde{Q}((z,y)\to (\bar{z},\bar{y}))$ on the state space $\tilde{Z}$. The corresponding acceptance probability for transition (*z*, *y*) → $\left(\bar{z},\bar{y}\right)$ is then given by

$$a((z,y)\to (\bar{z},\bar{y})):=\min\left\{1,\frac{\pi (\bar{z})p(\bar{y}|\bar{z})\tilde{Q}((\bar{z},\bar{y})\to (z,y))}{\tilde{Q}((z,y)\to (\bar{z},\bar{y}))\pi (z)p(y|z)}\right\}.$$

Using this Metropolis-Kernel defined on the enlarged state space $\tilde{Z}$, we construct a Markov chain ($\tilde{Z}$_*n*_) with invariant distribution *π*(*z*) and an initial state *z*_0 _on the original state space Z, as follows:

(i) given the previous state *z*_*n*_, sample *y*_*n *_~ *p*(*y*_*n*_|*z*_*n*_);

(ii) sample (*z'*, *y'*) ~ $\tilde{Q}$((*z*_*n*_, *y*_*n*_) → (*z'*, *y'*));

(iii) with probability *a*((*z*_*n*_, *y*_*n*_) → (*z'*, *y'*)) take *Z*_*n*+1 _= *z'*, otherwise *Z*_*n*+1 _= *z*_*n*_. Forget *y*_*n *_and *y'*.

Alternatively one could define a proposal kernel *Q*(*z *→ $\bar{z}$) directly on the original state space Z by summing out *y*, i.e.,

$$Q(z\to \bar{z}):=\sum_{y,\bar{y}\in Y}p(y|z)\tilde{Q}((z,y)\to (\bar{z},\bar{y})).$$

However, the computation of this transition distribution requires an extra summation step and there are situations in which we cannot afford using this direct proposal distribution in the Metropolis step.

### A.4 Constructing an initial configuration for the Markov chain

Now we return to our application. By using the Metropolis-Hastings algorithm, we shall construct a Markov chain on the configuration space Ω with invariant distribution equal to the constrained distribution (1).

Before entering that topic, however, we need to construct a configuration *ω*_0 _∈ C serving as an initial state for the Metropolis algorithm. In other words, we need to find an ancestral graph and corresponding gene flow variables that are logically consistent with the data. Since this is a nontrivial problem, we describe the procedure in detail. Later we use a similar construction to obtain a proposal distribution for the Metropolis algorithm.

We start from generation 0 with *n*(0) sampled individuals and their (partially) observed genotypes (*g*_*k*_(*l*) : *k *= 1, ..., *n*(0), *l *= 1, ..., *L*). Sequentially, following a uniformly distributed permutation, these individuals will choose their parents from generation 1 and transmit their genes to the chosen parents. Denote by {*X*_*k *_= (*f*, *m*)} the event that child *k *has chosen *f *as the father and *m *as the mother from amongst ${N^{\prime }}_{1}$ possible fathers and ${N^{\prime \prime }}_{1}$ possible mothers in the population. To start the construction, suppose that the first child chooses the first father *f *and the first mother *m *from the population, and transmits his/her alleles to these parents. For a generic locus *l*, let *g*_1_(*l*) = {*a*, *b*} be the genotype of the first child. With probability $\frac{1}{2}$ we set *h*_1_(*l*) = *a*, *h*_2_(*l*) = *b*, *g*_*f*_(*l*) = {*a*, ∅} and *g*_*m*_(*l*) = {*b*, ∅}, and otherwise we set *h*_1_(*l*) = *b*, *h*_2_(*l*) = *a*, *g*_*f *_(*l*) = {*b*, ∅} and *g*_*m*_(*l*) = {a, ∅}. Note that this determines only the haplotypes of the first child, and partially the genotypes of his/her parents, but does not give any information about the pattern of meioises that led to the haplotype of the child.

Proceeding recursively, assume that the first (*k *- 1) children in the present generation have already chosen altogether *F*(*k *- 1) fathers and *M*(*k *- 1) mothers, and have transmitted their alleles to the chosen parents. Child *k *can then choose parents from among these *F*(*k *- 1) fathers and *M*(*k *- 1) mothers or from the (${N^{\prime }}_{1}$ - *F*(*k *- 1)) fathers and (${N^{\prime \prime }}_{1}$ - *M*(*k *- 1)) mothers who had not yet been chosen by any child. In doing so the child must take into account his/her own genotype and the possibly censored genotypes of the candidate parents, which, at this stage, contain the alleles transmitted by the preceding (*k *- 1) children. Indeed child *k *chooses father *f *and mother *m *from generation 1 by sampling from a distribution proportional to

$$P(X_{k}=(f,m)|X_{1},...,X_{k-1})\prod_{l=1}^{L}P(g_{k}(l)|g_{f}(l),g_{m}(l)).$$

Here the term *P*(*X*_*k *_= (*f*, *m*)|*X*_1_, ..., *X*_*k*-1_) is the contribution from the prior distribution of the ancestral graph, *g*_*k*_(*l*) is the observed genotype (at locus *l*) of child *k*, and *g*_*f*_(*l*) and *g*_*m*_(*l*) are the current values of the partially determined genotypes of the parents. Finally, the transmission likelihood *P*(*g*_*k*_(*l*)|*g*_*f*_(*l*), *g*_*m*_(*l*)) is defined as follows under the assumption of free recombination.

If both parental genotypes *g*_*f*_(*l*) = {*a*, *b*} and *g*_*m*_(*l*) ={*c*, *d*}, with *a*, *b*, *c*, *d *∈ *E*_*l*_, are already fully determined,

$$\begin{array}{c} P(g_{k}(l)=\{x,y\}|g_{f}(l)=\{a,b\},g_{m}(l)=\{c,d\})\\ =\frac{1}{4}\{1(\{x,y\}=\{a,c\})+1(\{x,y\}=\{a,d\})+\\ 1(\{x,y\}=\{b,c\})+1(\{x,y\}=\{b,d\})\}. \end{array}$$

If some of the parental alleles are only partially determined by the previous transmission events, we integrate the missing alleles out:

$$\begin{array}{ll} & P(g_{k}(l)=\{x,y\}|g_{f}(l)=\{a,b\},g_{m}(l)=\{c,d\})\\ = & \sum_{a^{\prime },b^{\prime },c^{\prime },d^{\prime }\in E_{l}}\{P(g_{k}(l)=\{x,y\}|g_{f}(l)=\{a^{\prime },b^{\prime }\},g_{m}(l)=\{c^{\prime },d^{\prime }\})\times \\ & fr(\{a^{\prime },b^{\prime }\}|\{a,b\})\;fr(\{c^{\prime },d^{\prime }\}|\{c,d\})\},\;x,y\in E_{l},\;a,b,c,d\in E_{l}\cup \{\emptyset \}, \end{array}$$

where for *a*, *b*, *c*, *d *∈ *E*_*l*_, we define the conditional population genotype frequencies as

$$\begin{array}{c} fr(\{a,b\}|\{\emptyset ,\emptyset \})=fr(\{a,b\}),\\ fr(\{a,b\}|\{c,\emptyset \})=\frac{\left(1(c=a)+1(c=b))fr(\{a,b\}\right)}{2fr(\{c,\emptyset \})}\\ fr(\{a,b\}|\{c,d\})=1(a=c)1(b=d)+1(a\neq b)1(a=d)1(b=c). \end{array}$$

After choosing parents *f *and *m*, child *k *transmits his/her alleles to them. For that, let us consider the assignment of phase. Let *g*_*f*_(*l*) = {*a*, *b*} and *g*_*m*_(*l*) = {*c*, *d*} be the current values of the genotypes of the chosen parents with *a*, *b*, *c*, *d *∈ *E*_*l *_∪ {∅}, and let {*x*, *y*} be the genotype of the child. When *a*, *b*, *c*, *d *∈ *E*_*l *_are already determined, we have

$$\begin{array}{l} P(x\text{ paternal},y\text{ maternal}|g_{k}(l)=\{x,y\},g_{f}(l)=\{a,b\},g_{m}(l)=\{c,d\})\\ =\frac{P(g_{k}(l)=\{x,y\},x\text{ paternal},y\text{ maternal}|g_{f}(l)=\{a,b\},g_{m}(l)=\{c,d\})}{P(g_{k}(l)=\{x,y\}|g_{f}(l)=\{a,b\},g_{m}(l)=\{c,d\})} \end{array},$$

where

$$\begin{array}{c} \begin{array}{c} P(g_{k}(l)=\{x,y\},x\text{ paternal},y\text{ maternal}|g_{f}(l)=\{a,b\},g_{m}(l)=\{c,d\})\\ =\frac{1}{4}\{(1-\frac{1}{2}1(a=c))1(x=a)1(y=c)+(1-\frac{1}{2}1(a=d))1(x=a)1(y=d)+ \end{array}\\ \left(1-\frac{1}{2}1(b=c))1(x=b)1(y=c)+(1-\frac{1}{2}1(b=d))1(x=b)1(y=d)\right\} \end{array}$$

and then set, according to this probability, *h*_2*k*-1_(*l*) = *x *and *h*_2*k*_(*l*) = *y*, and otherwise *h*_2*k*-1_(*l*) = *y *and *h*_2*k*_(*l*) = *x*.

If there are undetermined alleles among *a*, *b*, *c *and *d*, we integrate them out with respect to the conditional genotype frequencies. Thus the parental origins of the alleles *x *and *y *are sampled according to the probability (10), where formula (11) is extended to the case of partially censored parental genotypes {*a*, *b*}, {*c*, *d*}, with *a*, *b*, *c*, *d *∈ *E*_*l *_∪ {∅} by

$$\begin{array}{ll} & P(g_{k}(l)=\{x,y\},x\text{ paternal},y\text{ maternal}|g_{f}(l)=\{a,b\},g_{m}(l)=\{c,d\})\\ = & \frac{1}{4}\sum_{a^{\prime },b^{\prime },c^{\prime }d^{\prime }\in E_{l}}\{[(1-\frac{1}{2}1(a^{\prime }=c^{\prime }))1(x=a^{\prime })1(y=c^{\prime })+\\ & (1-\frac{1}{2}1(a^{\prime }=d^{\prime }))1(x=a^{\prime })1(y=d^{\prime })+(1-\frac{1}{2}1(b^{\prime }=c^{\prime }))1(x=b^{\prime })1(y=c^{\prime })+\\ & (1-\frac{1}{2}1(b^{\prime }=d^{\prime }))1(x=b^{\prime })1(y=d^{\prime })]\;fr(\{a^{\prime },b^{\prime }\}|\{a,b\})\;fr(\{c^{\prime },d^{\prime }\}|\{c,d\})\}. \end{array}$$

Then, given the phase of the alleles of the child at locus *l*, we update independently the genotypes of the parents. To do so, consider the case where *h*_2*k*-1_(*l*) = *x*, that is, *x *came from the father, and let *g*_*f*_(*l*) = {*a*, *b*} be the current value of the father's genotype. There are three cases to consider: (i) If *a *and *b *are both determined, there is nothing to do, (ii) If *a *= *b *= ∅, we set *g*_*f*_(*l*) = {*x*, ∅}, and (iii) If *a *∈ *E*_*l *_and *b *= ∅, and if *a *≠ *x*, then the genotype of the father must be *g*_*f*_(*l*) = {*a*, *x*}, whereas if *a *= *x*, we set *g*_*f*_(*l*) = {*a*, *a*} with probability

$$\frac{fr(\{a,a\})}{fr(\{a,\emptyset \})+fr(\{a,a\})}$$

and otherwise leave *g*_*f*_(*l*) = {*a*, ∅}. After having completed this step for all the individuals in generation 0, we have determined their haplotypes and also partially the genotypes of their parents.

For the induction step, we assume that we have followed the procedure for *t *- 1 generations. and we now describe the procedure for generation *t *<*T*. The individuals in generation *t *have to choose parents from generation (*t *+ 1) and transmit their ancestral alleles to these parents. Note that the situation in generation *t > *0 differs from the situation in generation 0, since not all alleles are necessarily ancestral. Let *g*_*k*_(*l*) = {*a*, *b*} be the genotype of individual *k *in generation *t*. He or she will choose parents according to the distribution given in (8). In case *a*, *b *∈ *E*_*l*_, that is, both alleles are ancestral, we proceed as in the case *t *= 0. Otherwise, however, we need to specify the probabilities *P*(*g*_*k*_(*l*)|*g*_*f *_(*l*), *g*_*m*_(*l*)) also in cases in which *g*_*k*_(*l*) is censored or partially censored.

If *g*_*k*_(*l*) is completely censored, we make the convention that *P*(*g*_*k*_(*l*) = {∅, ∅}|*g*_*f*_(*l*), *g*_*m*_(*l*)) ≡ 1. If *g*_*k*_(*l*) is partially censored, we define for *x *∈ *E*_*l *_and *a*, *b*, ∈ *E*_*l *_∪ {∅},

$$fr(x|\{a,b\}):=fr(\{x,\emptyset \}|\{a,b\}):=\frac{1}{2}\sum_{a^{\prime },b^{\prime }\in E_{l}}\left(1(x=a^{\prime })+1(x=b^{\prime }))fr(\{a^{\prime },b^{\prime }\}|\{a,b\}\right),$$

which is the conditional probability of picking the allele *x *from the partially observed genotype pair {*a*, *b*} that was sampled from the population. Then we define, for *x*, *a*, *b*, *c*, *d *∈ *E*_*l*_,

$$P(g_{k}(l)=\{x,\emptyset \}|g_{f}(l)=\{a,b\},g_{m}(l)=\{c,d\})=\frac{1}{2}fr(\{x,\emptyset \}|\{a,b\})+\frac{1}{2}fr(\{x,\emptyset \}|\{c,d\}),$$

which is the probability that, given the information on the genotypes of the parents, a randomly chosen allele from the genotype of child *k *at locus *l *is of type *x*. Having chosen the parents, we decide the parental origin of the allele *x *according to the probability

$$P(x\text{ paternal}|g_{k}(l)=\{x,\emptyset \},g_{f}(l)=\{a,b\},g_{m}(l)=\{c,d\})=\frac{fr(\{x,\emptyset \}|\{a,b\})}{fr(\{x,\emptyset \}|\{a,b\})+fr(\{x,\emptyset \}|\{c,d\})}.$$

After this, it remains to update the genotype of the chosen parent by transmitting the allele *x*. This is done in exactly the same way as for the alleles in generation 0.

Having followed this procedure for all children in generation *t*, we have determined their ancestral haplotypes, the censoring pattern on these haplotypes, and the ancestral genotypes of the parents. Moreover, when *t *> 0, this determines partially the meiosis pattern of the ancestral haplotypes of the individuals in generation (*t *- 1). For example, let *m *be the mother (in generation *t*) of child *k *(in generation (*t *- 1)). This child has inherited from his/her mother the haplotype *h*_2*k*_, and the corresponding meiosis pattern *ψ*_2*k *_is determined, for every locus *l*, as follows:

(i) If *h*_2*k*_(*l*) = ∅, we set *ψ*_2*k*_(*l*) = ∅.

(ii) Let *h*_2*k*_(*l*) = *x *∈ *E*_*l*_, and let the alleles of the mother, respectively of grandpaternal and grandmaternal origin, be *h*_2*m*-1_(*l*) = *a *and *h*_2*m*_(*l*) = *b*, *a*, *b *∈ *E*_*l *_∪ {∅}. Note that (*a*, *b*) ≠ (∅, ∅), since either (*a *= *x*) or (*b *= *x*). If *a *≠ *b*, the grandparental origin of the ancestral allele *x *is determined as *ψ*_2*k*_(*l*) = 1(*x *= *a*). The grandparental origin of *x *remains undetermined only when *a *= *b *= *x *∈ *E*_*l*_. In such a case we have to sample simultaneously the grandparental origins of all ancestral alleles at locus *l *in generation *t *- 1 that are inherited from the mother *m*. In order to do so, suppose that *k*_1_, ..., *k*_*n *_are the children of mother *m *and *h*_2*m*-1_(*l*) = *h*_2*m*_(*l*) = $h_{2k_{1}}(l)=...=h_{2k_{n}}(l)$ = *x *∈ *E*_*l*_. Note that since the mother has two ancestral alleles, necessarily *n *≥ 2, and we may exclude the event $I=\{\psi_{2k_{1}}(l)=\psi_{2k_{2}}(l)=...=\psi_{2k_{n}}(l)\}$.

Conditioning on the complementary event *I*^*c *^is equivalent to the following procedure: We sample without replacement two children, say $\hat{k}$ and $\bar{k}$, among {*k*_1_, ..., *k*_*n*_}, and assign $\psi_{2\hat{k}}$(*l*) = 0 and $\psi_{2\bar{k}}$(*l*) = 0. Given this the grandparental origins of the remaining children are conditionally independent Bernoulli ($\frac{1}{2}$) random variables.

We iterate the above procedure backwards in time until we reach the founder generation, where we determine, by tossing fair coins, the parental origins of the founders' ancestral genotypes. Given that, we compute the meiosis pattern on the haplotypes in generation (*T *- 1) as described above.

#### Remarks

(1) Although at a first reading these sampling formulae may not be completely obvious, the idea behind the sequential scheme is simple: a child chooses his/her parents from the population and transmits his/her alleles to the parents according to Bayes' formula, by conditioning on his/her own alleles as well as on the parental choices and allele transmissions of the previous children. Note, however, that the procedure is not fully Bayesian, since the conditioning does not include at every intermediate stage the information about the genotypes of the children still in the list, and that we are not taking into account the true recombination likelihood. Therefore the resulting configuration is only compatible with the data, but not an exact sample from the posterior distribution.

(2) An alternative way to proceed would be to first let all children from the considered generation choose their parents, and then sample jointly the parental phases of the alleles of the children given the family structure, and finally transmit the alleles to the parents. We develop this idea later in section A.7, as we construct a block-update for the Metropolis-Hastings algorithm.

(3) If the population of candidate parents is small, it is possible that, when a child *k *is choosing his/her parents, the genotypes of the possible parents are already partially determined by the alleles of the previous (*k *- 1) children in such a way that every possible parental choice is logically incompatible with the genotype of child *k*. When such a contradiction is found, we have to restart from the beginning, or at least from one generation back. If the algorithm keeps failing, we may need to increase the size of the population in the prior.

#### A.4.1 Incorporating the true recombination likelihood

In the construction of an initial configuration we have used the model with free recombination. In the case of closely linked markers, the resulting initial configuration will be compatible with the data, but it will not look realistic, since most likely it will contain too many recombinations.

It is shown in Appendix B, how to apply the Viterbi algorithm to sample the parental phase vector *ϕ*_*k *_= (*ϕ*_*k*_(1), ..., *ϕ*_*k*_(*L*)) of an individual *k*, say for a female, in generation *t *> 0, from a joint conditional distribution, where we condition on her partially observed genotypes (*g*_*k*_(1), ..., *g*_*k*_(*L*)), on the partially observed genotypes (*g*_*f*_(1), ..., *g*_*f*_(*L*)) and (*g*_*m*_(1), ..., *g*_*m*_(*L*)) of her parents *f *and *m *in generation (*t *+ 1), and on the partially observed haplotypes {($h_{2k(j)}(1),...,h_{2k(j)}(L))$ : *j *= 1, ..., *n*} which she has transmitted to her children *k*(1), ..., *k*(*n*) in generation (*t *- 1). We can then generally improve on the initial configuration by substituting the sampling distribution (10) or (11) by this joint conditional distribution that takes into account the true recombination likelihood of the haplotypes of the children. It is also shown in Appendix B, how to integrate out the phase vector *ϕ*_*k*_, in order to compute the marginal likelihood of the partially observed haplotypes of the children. We could compute this marginal likelihood for all logically compatible choices of pairs of grandparents and include it as a new factor in expression (8) which is proportional to the probability of choosing a pair of grandparents. Alternatively, we could choose the grandparents using expression (8) with free recombination, and then use the true recombination likelihood only for assigning the parental origins to the genes of the parent.

### A.5 Block-updates for the Metropolis-Hastings algorithm

Next we discuss the construction of a proposal distribution *Q*(*ω *→ $\bar{\omega }$) for the Metropolis-Hastings algorithm on the configuration space Ω. Ideally, we would like to propose only configurations that are compatible with the data, or at least we want that *Q*(*ω *→ C) is not too small when starting from some *ω *∈ C. The resulting Markov chain should also be irreducible, that is, it should be able to reach every state in C with positive probability regardless of where it started from. Single-site updates, like changing the phase of one allele at a time, are not enough to produce an irreducible chain. Larger block-updates are needed also because changes to the ancestral graph usually require simultaneous changes to the allelic paths.

Our aim is to construct a block-proposal distribution by applying locally similar ideas that were used in generating an initial configuration. Starting from some configuration *ω *∈ C, a selected group of individuals in the ancestral graph try to choose new parents and redirect their ancestral alleles from the "old ancestors" to the new ones. This is done by conditioning on the paths of the ancestral alleles of the other individuals, which are kept fixed during the block-update step. Before making these points more precise, we introduce some more notation.

#### A.5.1 Conditional probabilities for the types of non-ancestral alleles

Given a configuration *ω*, we define *fr*(*a*|*k*, *ω*) for any locus *l *and individual *k *as the conditional probability that *k *transmits an allele of type *a *∈ *E*_*l *_at locus *l *to a "hypothetical new child". When the genotype *g*_*k *_= {$g_{k}^{0},g_{k}^{1}$} at locus *l *of individual *k *is fully ancestral, we have

$$fr(a|k,\omega )=\frac{1}{2}\{1(a=g_{k}^{0})+1(a=g_{k}^{1})\}.$$

Otherwise individual *k *has at least one non-ancestral allele at this locus, and in order to compute *fr*(*a*|*k*, *ω*), we must take into account all possible ways in which he/she may have inherited the non-ancestral allele(s) from his/her ancestors.

We also need the joint transmission probabilities for pairs of alleles. We denote by *fr*(*a*, *a'*|*k*, *k'*, *ω*) the conditional probability that individual *k *transmits an allele of type *a *to his/her hypothetical new child and simultaneously individual *k' *transmits an allele of type *a' *to his/her hypothetical new child. Here *a*, *a' *∈ *E*_*l*_, and we let the indexes *k, k' *go through all individuals in a given generation. If at least one of the genotypes *g*_*k *_or *g*_*k' *_is fully ancestral,

*fr*(*a*, *a'*|*k*, *k'*, *ω*) = *fr*(*a*|*k*, *ω*)*fr*(*a'*|*k'*, *ω*).

However, this equation may not hold, if in configuration *ω *both *k *and *k' *carry some non-ancestral allele at locus *l*, and there is a common ancestor of *k *and *k' *who has had a positive probability of transmitting the same non-ancestral allele to both *k *and *k'*.

We compute these transmission probabilities recursively, starting from the founders, by setting

*fr*(*a*|*k*, *ω*) = *fr*(*a*|*g*_*k*_),

where *g*_*k *_= {$g_{k}^{0},g_{k}^{1}$} ⊆ *E*_*l *_∪ {∅}, is the possibly censored genotype of founder *k *in configuration *ω*, and *fr*(*a*|{*x*, *y*}) was defined in (12) by using the genotype frequencies of the population. Moreover, if *k *and *k' *are distinct founders, we have

*fr*(*a*, *a'*|*k*, *k'*, *ω*):= *fr*(*a*|*k*, *ω*)*fr*(*a'*|*k'*, *ω*),

whereas for *k *= *k'*

$$fr(a,a^{\prime }|k,k,\omega ):=\sum_{\left\{x,y\right\}}fr(a|\{x,y\})fr(a^{\prime }|\{x,y\})fr(\{x,y\}|\{g_{k}^{0},g_{k}^{1}\}),$$

where the sum is taken over all possible genotypes at locus *l*. It remains to specify *fr*(*a*|*k*, *ω*) and *fr*(*a*, *a'*|*k*, *k'*, *ω*), when *k *and *k' *are not founders and *g*_*k *_or ${g^{\prime }}_{k}$ contains non-ancestral alleles. For that let *f *and *m *be the father and the mother of *k*, and let *x *and *y *be his/her possibly censored alleles at locus *l *of paternal and maternal origin, respectively. Since we proceed recursively, we may assume that *fr*(*a*|*f*, *ω*) and *fr*(*a*|*m*, *ω*) are already computed. Then

*fr*(*a*|*k*, *ω*) = $\frac{1}{2}$ {1(*x *= *a*) + 1(*x *= ∅)*fr*(*a*|*f*, *ω*) + 1(*y *= *a*) + 1(*y *= ∅)*fr*(*a*|*m*, *ω*)},

for all *a *∈ *E*_*l*_. It remains to speficy *fr*(*a*, *a'*|*k*, *k'*, *ω*), when *k *and *k' *are not founders and both genotypes *g*_*k *_= {*x*, *y*} and ${g^{\prime }}_{k}$ = {*x'*, *y'*} contain some non-ancestral alleles. Let (*f*, *m*) and (*f'*, *m'*) be the parents of *k *and *k'*, and assume that *x*, *x' *∈ *E*_*l *_∪ {∅} are paternal and *y*, *y' *∈ *E*_*l *_∪ {∅} are maternal. For *a*, *a' *∈ *E*_*l*_, we obtain, again using recursion, that

$$\begin{array}{l} fr(a,a^{\prime }|k,k^{\prime },\omega )=\frac{1}{4}(\left\{1(x=a)+1(y=a)\}\{1(x^{\prime }=a^{\prime })+1(y^{\prime }=a^{\prime })\right\}\\ +\{1(x,\emptyset )fr(a|f,\omega )+1(y=\emptyset )fr(a|m,\omega )\}\{(1(x^{\prime }=a^{\prime })+1(y^{\prime }=a^{\prime })\}\\ +\{(1(x=a)+1(y=a)\}\{1(x^{\prime }=\emptyset )fr(a^{\prime }|f^{\prime },\omega )+1(y^{\prime }=\emptyset )fr(a^{\prime }|m^{\prime },\omega )\}\\ +1(x=\emptyset )1(x^{\prime }=\emptyset )fr(a,a^{\prime }|f,f^{\prime },\omega )+1(x=\emptyset )1(y^{\prime }=\emptyset )fr(a,a^{\prime }|f,m^{\prime },\omega )\\ +1(y=\emptyset )1(x^{\prime }=\emptyset )fr(a,a^{\prime }|m,f^{\prime },\omega )+1(y=\emptyset )1(y^{\prime }=\emptyset )fr(a,a^{\prime }|m,m^{\prime },\omega )), \end{array}$$

where the formula holds also if *k *= *k' *or *f *= *f' *or *m *= *m'*.

These recursive formulae are exact, taking into account all intersections between the possible paths of the two non-ancestral alleles. In principle we could extend these formulae to allele triples, quadruples, and generally, for *n*-tuples. However, since we will consider only one genotype at a time, we need only the joint transmission probabilities for pairs of alleles.

#### A.5.2 Conditional genotype probabilities

For any pair of candidate parents *f *and *m*, belonging to generation 1 ≤ *t *≤ *T*, we define the conditional genotype probabilities at every locus *l *of a hypothetical common child *k *by

*P*({*a*, *b*}|*f*, *m*, *ω*) := *fr*(*a*, *b*|*f*, *m*, *ω*) + 1(*a *≠ *b*)*fr*(*b*, *a*|*f*, *m*, *ω*), *a*, *b *∈ *E*_*l*_.

We also define, for *a *∈ *E*_*l*_,

$$\begin{array}{l} P(\{a,\emptyset \}|f,m,\omega ):=fr(a|f,m,\omega ):=\frac{1}{2}fr(a|f,\omega )+\frac{1}{2}fr(a|m,\omega )\\ =\frac{1}{2}\sum_{b\in E_{l}}\left\{fr(a,b|f,m,\omega )+fr(b,a|f,m,\omega )\right\}\\ =\frac{1}{2}\left\{fr(\{a,a\}|f,m,\omega )+\sum_{b\in E_{l}}fr(\{a,b\}|f,m,\omega )\right\}, \end{array}$$

which corresponds to the conditional probability that an allele at locus *l *of a hypothetical common child is of type *a*. We also set, as a convention, *P*({∅, ∅}|*f*, *m*, *ω*) = 1.

### A.6 Block-update I: Children choosing new parents

We consider a randomly selected group of children, indexed by *k*_1_, ..., *k*_*n*_, all belonging to the same generation 0 ≤ *t *<*T*, who will choose new parents and redirect their ancestral alleles from their "old ancestors" to "new ancestors". Before choosing new parents, these children must withdraw their alleles from their old parents. Starting from configuration *ω*, we construct a modified configuration $\bar{\omega }$ in which, ascending from generation (*t *+ 1) to generation *T*, we delete the paths of the ancestral alleles that go through the children *k*_1_, ..., *k*_*n*_. Consequently all the ancestral alleles that were transmitted only by these children become censored.

Next we look at the individuals in generation (*t *+ 1), including the part of the population which was left outside the ancestral graph, and consider all possible parental pairs (*f*, *m*). Recall that the individuals outside the ancestral graph have genotype {∅, ∅} by default, and their conditional genotype probabilities coincide with the population genotype frequencies. Following the sequential ordering, each of the children *k*_1_, ..., *k*_*n *_will choose randomly a pair of parents from generation (*t *+ 1) and transmit to them (and to their ancestors) his/her ancestral alleles. Locally this is like the construction of the initial configuration explained in section A.4, with the difference that here we must condition on the upper part of the ancestral graph and on the allelic paths that are determined by the modified configuration $\bar{\omega }$. Indeed, if child *k *has genotypes $\left\{g_{k}^{0}(l),g_{k}^{1}(l)\right\}$, then he/she chooses parental pair *X*_*k *_= (*f*, *m*) with a probability proportional to expression (8), where we extend the definition of *P*(*g*_*k*_(*l*)|*g*_*f*_(*l*), *g*_*m*_(*l*)) to non-founder parents by means of the conditional genotype probabilities *P*(*g*_*k*_(*l*)|*f*, *m*, $\bar{\omega }$) given in formulae (13) and (13).

#### A.6.1 Dropping and adding ancestors

After the children *k*_1_, ..., *k*_*n *_have chosen new parents, it is possible that a former parent in the ancestral graph is left without children. In this case he or she will be dropped from the ancestral graph. By induction, the same may apply to the more distant ancestors in the elder generations. In order to obtain a reversible Markov chain, the children are given the possibility of choosing new parents also from the population outside the ancestral graph, whence these new parents together with their ancestors will become a part of the updated ancestral graph.

Note that it is straightforward to use the sequential construction of the prior distribution to sample the ancestry of the population outside the ancestral graph, conditionally on the ancestral graph. In principle such a resampling step must be included in the MCMC algorithm as a pre-move, before updating the ancestral graph. However, that may not be practical if the size of the population is large. In that case, instead of resampling the ancestry of all members of the population, it is enough to sample (conditionally on the current ancestral graph) the ancestry of a limited number of candidate parents outside the ancestral graph.

#### A.6.2 Resampling the paths of the ancestral alleles

Having chosen a new father *f *and a new mother *m *for child *k*, we sample new parental origins of the ancestral alleles of *k *by extending formulae (10) and (11) as follows: When both $g_{k}^{0}$(*l*) and $g_{k}^{1}$(*l*) are ancestral, we have

$$P(g_{k}^{0}(l)\text{ paternal},g_{k}^{1}(l)\text{ maternal}|g_{k}(l),f,m,\bar{\omega })=\frac{fr(g_{k}^{0}(l),g_{k}^{1}(l)|f,m,\bar{\omega })}{fr(g_{k}^{0}(l),g_{k}^{1}(l)|f,m,\bar{\omega })+fr(g_{k}^{1}(l),g_{k}^{0}(l)|f,m,\bar{\omega })}.$$

Otherwise, if $g_{k}^{0}$(*l*) is ancestral and $g_{k}^{1}$(*l*) is non-ancestral, we have

$$P(g_{k}^{0}(l)\text{ paternal},g_{k}^{1}(l)\text{ maternal}|g_{k}(l),f,m,\bar{\omega })=\frac{fr(g_{k}^{0}(l)|f,\bar{\omega })}{fr(g_{k}^{0}(l)|f,\bar{\omega })+fr(g_{k}^{0}(l)|m,\bar{\omega })},$$

and symmetrically, if $g_{k}^{1}$(*l*) is ancestral and $g_{k}^{0}$(*l*) is non-ancestral,

$$P(g_{k}^{0}(l)\text{ paternal},g_{k}^{1}(l)\text{ maternal}|g_{k}(l),f,m,\bar{\omega })=\frac{fr(g_{k}^{1}(l)|m,\bar{\omega })}{fr(g_{k}^{1}(l)|f,\bar{\omega })+fr(g_{k}^{1}(l)|m,\bar{\omega })}.$$

Finally, if both $g_{k}^{0}$(*l*) and $g_{k}^{1}$(*l*) are non-ancestral, we have that

$$P(g_{k}^{0}(l)\text{ paternal},g_{k}^{1}(l)\text{ maternal}|g_{k}(l),f,m,\bar{\omega })=\frac{1}{2}.$$

After this, in case one or two ancestral alleles were transmitted, we must update the genotypes of the new parents *f *and *m*, and eventually also the genotypes of their ancestors higher up in the ancestral graph. If both genotypes of the new parents are already fully ancestral at the given locus *l*, there is nothing to do. If the parents are founders, we proceed as in section A.4. Otherwise, let the alleles in locus *l *of child *k *be *h*_2*k*-1_(*l*) = *a *(paternal) and *h*_2*k*_(*l*) = *b *(maternal), with *a*, *b *∈ *E*_*l *_∪ {∅}, and at least one of them ancestral. Consider first the case in which only one of *a *and *b *is ancestral and, for example, that it is paternal. If the father *f *has already two ancestral alleles, there is nothing to do. Otherwise, let *h*_2*f*-1_(*l*) = *x *and *h*_2*f*_(*l*) = *y*, *x*, *y *∈ *E*_*l *_∪ {∅}, where *x *or *y *or both are non-ancestral. We denote by *f' *and *m' *the father and the mother of *f*, respectively. With probability

$$\frac{1(x=a)+1(x=\emptyset )fr(a|f^{\prime },\bar{\omega })}{1(x=a)+1(x=\emptyset )fr(a|f^{\prime },\bar{\omega })+1(y=a)+1(y=\emptyset )fr(a|m^{\prime },\bar{\omega })}$$

the ancestral allele *a *was inherited from the grandfather *f'*, and in case *h*_2*f*-1_(*l*) was censored, we update it to *h*_2*f*-1_(*l*) = *a*, and leave *h*_2*f*_(*l*) = *y*. Otherwise *a *was inherited from the grandmother *m'*, and if *h*_2*f*_(*l*) was censored, we update it by setting *h*_2*f*_(*l*) = *a*, and leave *h*_2*f*-1_(*l*) = *x*.

Next we consider the case where both *a *and *b *are ancestral. If only one parent has censored alleles, we are back to the previous case, since at most one parental allele will be updated. Otherwise we have to follow the origins of *a *and *b *simultaneously. Assume therefore that both *f *and *m *have censored alleles, that is, *h*_2*f*-1_(*l*) = *x*, *h*_2*f*_(*l*) = *y *and *h*_2*m*-1_(*l*) = *x'*, *h*_2*m*_(*l*) = *y'*, with *x *or *y *or both censored and *x' *or *y' *or both censored. Let *f' *and *m' *be the father and mother of *f*, and let *f" *and *m" *be the father and mother of *m*. Then with probability

$$\begin{array}{l} \left\{1(x=a)1(x^{\prime }=b)+1(x=a)1(x^{\prime }=\emptyset )fr(b|f^{\prime \prime },\bar{\omega }\right)\\ +1(x=\emptyset )1(x^{\prime }=b)fr(a|f^{\prime },\bar{\omega })+1(x=\emptyset )1(x^{\prime }=\emptyset )fr(a,b|f^{\prime },f^{\prime \prime },\bar{\omega })\}/C \end{array}$$

*a *was inherited from *f' *and *b *was inherited from *f"*, with probability

$$\begin{array}{l} \left\{1(y=a)1(x^{\prime }=b)+1(y=a)1(x^{\prime }=\emptyset )fr(b|f^{\prime \prime },\bar{\omega }\right)\\ +1(y=\emptyset )1(x^{\prime }=b)fr(a|m^{\prime },\bar{\omega })+1(y=\emptyset )1(x^{\prime }=\emptyset )fr(a,b|m^{\prime },f^{\prime \prime },\bar{\omega })\}/C \end{array}$$

*a *was inherited from *m' *and *b *was inherited from *f"*, with probability

$$\begin{array}{l} \left\{1(x=a)1(y^{\prime }=b)+1(x=a)1(y^{\prime }=\emptyset )fr(b|m^{\prime \prime },\bar{\omega }\right)\\ +1(x=\emptyset )1(y^{\prime }=b)fr(a|f^{\prime },\bar{\omega })+1(x=\emptyset )1(y^{\prime }=\emptyset )fr(a,b|f^{\prime },m^{\prime \prime },\bar{\omega })\}/C \end{array}$$

*a *was inherited from *f' *and *b *was inherited from *m"*, and finally with probability

$$\begin{array}{l} \left\{1(y=a)1(y^{\prime }=b)+1(y=a)1(y^{\prime }=\emptyset )fr(b|m^{\prime \prime },\bar{\omega }\right)\\ +1(y=\emptyset )1(y^{\prime }=b)fr(a|m^{\prime },\bar{\omega })+1(y=\emptyset )1(y^{\prime }=\emptyset )fr(a,b|m^{\prime },m^{\prime \prime },\bar{\omega })\}/C \end{array}$$

*a *was inherited from *m' *and *b *was inherited from *m"*. Here *C *is a normalizing constant. In each of these cases, the corresponding alleles of the parents become ancestral if they were non-ancestral before. This completes the updating procedure for the alleles of the parents.

If some censored allele became ancestral we must update the alleles of the grandparents as well, and possibly continue the procedure further backwards in time, until the alleles coalesce to some ancestral alleles or until the founder generation is reached. This is done in the same way as we updated the alleles of the parents.

We resume the updating procedure as follows. Given the choice of new parents, we sample new allelic paths for the ancestral alleles carried by the child. The path of an allele is a random walk on the ancestral graph, where in each generation the allele is assigned to either the paternal or the maternal origin, conditionally on the paths of the other ancestral alleles determined by the configuration $\bar{\omega }$. The new path of an ancestral allele is sampled sequentially until it crosses a path of an ancestral allele of the same type in the configuration $\bar{\omega }$, or until the path reaches the founder generation. Note also that, if the child transmits two ancestral alleles to the parents at some locus *l*, we are coupling the corresponding allelic paths in such a way that the paths are always compatible with each other and with the configuration $\bar{\omega }$.

#### A.6.3 Incorporating the true recombination likelihood

In the update procedure we have described so far, the transmission patterns are resampled by using the model with free recombination. However, we are able to take the true recombination likelihood partially into account. Namely, having assigned the new parents *f *and *m *to child *k *in generation 0 <*t *<*T*, we sample jointly the vector of the parental origins (*ϕ*_*k*_(1), ..., *ϕ*_*k*_(*L*)) of the alleles $\left(\{g_{k}^{0}(l),g_{k}^{1}(l)\},l=1,...,L\right)$ of *k*, by conditioning on the ancestral alleles of the ancestors of *k *and on the ancestral haplotypes that he/she transmits to his/her children in generation (*t *- 1). This is done by the Viterbi algorithm given in appendix B, where we specify the prior *π *for the vector of parental origins (*ϕ*_*k*_(1), ..., *ϕ*_*k*_(*L*)) as

$$\begin{array}{l} \pi (\phi_{k}(1),...,\phi_{k}(L))=\prod_{l=1}^{L}\pi_{k}(\phi_{k}(l),l),\text{ where}\\ \pi_{k}(0,l)=1-\pi_{k}(1,l)=P(g_{k}^{0}(l)\text{ paternal},g_{k}^{1}(l)\text{ maternal}|g_{k}(l),f,m,\bar{\omega }), \end{array}$$

and the right hand side of the last expression was defined in formulas (14–17).

Similarly we improve the procedure which updates the paths of the ancestral alleles (see section A.6.2). When an ancestor receives alleles from his/her descendants, some of his/her censored alleles may become ancestral and we need to sample the phases of these alleles. The Viterbi algorithm can be used to sample all these phases jointly (keeping the phases of the ancestral alleles fixed) by combining the product of the sampling distributions across the marker loci as given in section A.6.2 with the recombination likelihood contribution of the children's haplotypes.

#### A.6.4 Completing the block-update

Once the procedure is completed, we have updated the modified configuration $\bar{\omega }$ by assigning new parents to children *k*_1_, ..., *k*_*n *_and new paths to their ancestral alleles. The resulting updated configuration $\bar{\omega }$ is the proposal state in the Metropolis-Hastings algorithm. Note that when creating $\bar{\omega }$ we are also able to compute sequentially the proposal probability *Q*(*ω *→ $\bar{\omega }$), and similarly, starting from $\bar{\omega }$, we can compute the proposal probability *Q*($\bar{\omega }$ → *ω*) for the reverse transition. Therefore the corresponding Metropolis-Hastings update can be implemented.

We also use slightly different versions of this block-update. In the first of these the children do not change parents but the paths of their ancestral alleles are updated simultaneously. This is done by simply skipping the sampling of parents described in section A.6. In the other version we let the children involved in the update belong to different generations.

#### Remarks

(1) If one child is selected to choose new parents under the model with free recombination this proposal distribution is a Gibbs' update (see section A.2.1). The same does not hold more generally, since, when updating the parental choice and the paths of the ancestral alleles of child *k*_1_, we take into account neither the ancestral alleles of the other children *k*_2_, ..., *k*_*n*_, nor the complete recombination likelihood.

(2) If more than one child are selected for an update, it is possible that a selected child *k*_*i *_does not find any parents compatible with his/her genotypes. This may happen when the children *k*_1_, ..., *k*_*i*-1 _have already transmitted their alleles to their ancestors in such a way that it is no longer possible to extend the paths of all ancestral alleles of child *k*_*i *_up to the founder generation. In this case the proposed block-update is rejected.

### A.7 Block-update II: Half-siblings changing one parent

We take a random father in generation (*t *+ 1) ≤ *T *and consider all his children belonging to generation *t*, denoted by *k*_1_, ..., *k*_*n*_. These children are going to stay with their original father but will choose new mothers and consequently the paths of their ancestral alleles will be resampled. (To be politically correct, we also use the symmetric update which switches the roles of mothers and fathers.) We could continue as in the previous sections, resampling sequentially, for one child at a time, a new mother and new paths of the ancestral alleles. However, there is a potential problem here: as explained in section A.6.2, after a new mother has been chosen, the parental phases of the alleles of child *k*_1 _are resampled without simultaneously considering the ancestral alleles of children *k*_2_, ..., *k*_*n*_. When there are many children and many marker loci, it becomes unlikely that this procedure will assign several children to the same mother, and most of the time the algorithm proposes to add more mothers to the ancestral graph than would be necessary. As a consequence, if the true ancestral graph contains couples with many children, the corresponding Metropolis chain is slowly mixing, and the mixing gets even worse as the number of markers increases. To improve on that, we change the order in the resampling procedure. First the children *k*_1_, ..., *k*_*n *_choose new mothers, and then we sample jointly the new parental phases of their alleles, by conditioning on the ancestral alleles of the new parents and taking into account the recombination likelihood contribution from the haplotypes of the children *k*_1_, ..., *k*_*n*_. This block-update is computationally demanding, but it concerns only a small number of children at a time.

#### A.7.1 Sampling distribution for choosing the mothers

We start from the modified configuration $\bar{\omega }$ obtained as in section A.6 from the current configuration *ω *by withdrawing the ancestral alleles transmitted by the children *k*_1_, ..., *k*_*n *_to their ancestors. Let *f *be the common father of these children. Let *k*_1_ choose his/her mother as in section A.6, with the constraint that the father *f *is fixed. Thus *k*_1 _chooses his/her mother *m*_1 _from a distribution proportional to

$$P(X_{k_{1}}=(f,m)|X_{j},j\notin \{k_{1},...,k_{n}\})\prod_{l=1}^{L}P(g_{k_{1}}(l)|f,m,\bar{\omega }).$$

Instead of continuing as in section A.6.2, i.e. by sampling the parental phases of the alleles of *k*_1 _and updating the genotypes of the parents *f *and *m*_1 _at each locus *l*, we compute the joint conditional distribution

$$P(\phi_{k_{1}}(l),{\bar{g}}_{f}(l),{\bar{g}}_{m_{1}}(l)|g_{k_{1}}(l),f,m_{1},\bar{\omega })$$

under the model with free recombination. Here at every locus *l*, ${\bar{g}}_{f}$(*l*) and ${\bar{g}}_{m_{1}}$(*l*) are possible values of the updated genotypes of the parents after *k*_1 _has transmitted to them his/her ancestral alleles, and $\phi_{k_{1}}$(*l*) is the parental phase of the alleles of *k*_1_. The computation of (20) is done by combining the computations from section (A.6.2).

We shall illustrate the procedure with an example. Assume that given the configuration $\bar{\omega }$, the ancestral genotypes of the parents and the child are respectively *g*_*f *_(*l*) = {∅, ∅}, ${\bar{g}}_{m_{1}}$(*l*) = {*a*, ∅}, and $g_{k_{1}}$(*l*) = {*a*, *b*}. In this particular case formula (20) would give the following probabilities

$$\begin{array}{lll} p_{1} & \propto & P(f\text{ transmits }a|\bar{\omega })\times P(g_{m_{1}}(l)=\{a,b\}|m_{1}\text{ transmits }a,\bar{\omega }),\\ p_{2} & \propto & P(f\text{ transmits }b|\bar{\omega }),\\ p_{3} & \propto & P(f\text{ transmits }b|\bar{\omega })\times P(g_{m_{1}}=\{a,a\}|m_{1}\text{ transmits }a,\bar{\omega }). \end{array}$$

**Table 1:** 

$\phi_{k_{1}}$(*l*)	${\bar{g}}_{f}$(*l*)	${\bar{g}}_{m_{1}}$(*l*)	P
0	{*a*, ∅}	{*a*, *b*}	*p*_1_
1	{*b*, ∅}	{*a*, ∅}	*p*_2_
1	{*b*, ∅}	{*a*, *a*}	*p*_3_

Proceeding then by induction, suppose that children *k*_1_, ..., *k*_*i*-1 _have chosen mothers *m*_1_, ..., *m*_*M*(*i*-1)_, where *M*(*i *- 1) ≤ *i *- 1, and for each locus *l *we have computed the joint conditional distribution for the phases of the alleles of the children *k*_1_, ..., *k*_*i*-1 _and of the genotypes of the parents, denoted by

$$P(\phi_{k_{1}}(l),...,\phi_{k_{i-1}}(l),{\bar{g}}_{f}(l),{\bar{g}}_{m_{1}}(l),...,g_{m_{M(i-1)}}(l)|g_{k_{1}}(l),...,g_{k_{i-1}}(l),f,m_{1},...,m_{M(i-1)},\bar{\omega }).$$

Given that, we must specify how *k*_*i *_chooses his/her mother, and consequently, how the joint conditional distribution for the phases of the children's alleles and of the genotypes of the parents is updated.

Child *k*_*i *_will choose either one of the previously chosen mothers *m*_1_, ..., *m*_*M*(*i*-1)_, or a mother who was not yet chosen by any of the first (*i *- 1) children, with probability proportional to

$$P(X_{k_{i}}=(f,m)|X_{j},j\notin \{k_{i+1},...,k_{n}\})\times \prod_{l=1}^{L}P(g_{k_{i}}(l)|f,m,\bar{\omega },g_{k_{1}}(l),X_{k_{1}},...,g_{k_{i-1}}(l),X_{k_{i-1}}),$$

where *X*_*j *_= (*f*_*j*_, *m*_*j*_) denotes the parental choice of child *j*, and

$$\begin{array}{l} P(g_{k_{i}}(l)|f,m,\bar{\omega },g_{k_{1}}(l),X_{k_{1}},...,g_{k_{i-1}}(l),X_{k_{i-1}})\\ =\sum_{g_{f},g_{m}}P(g_{k_{i}}(l)|g_{f}(l),g_{m}(l))P(g_{f}(l),g_{m}(l)|\bar{\omega },g_{k_{1}}(l),X_{k_{1}},...,g_{k_{i-1}}(l),X_{k_{i-1}}). \end{array}$$

In the last expression the ancestral genotypes of the father and the mother are integrated out with respect to the conditional probability (20), after it is extended to the case in which a mother *m *had not yet been chosen by any of the first (*i *- 1) children, by the formula

$$P(g_{f}(l),g_{m}(l)|\bar{\omega },g_{k_{1}}(l),X_{k_{1}},...,g_{k_{i-1}}(l),X_{k_{i-1}})=P(g_{f}(l)|\bar{\omega },g_{k_{1}}(l),X_{k_{1}},...,g_{k_{i-1}}(l),X_{k_{i-1}})\times P(g_{m}(l)|\bar{\omega }).$$

Continuing with the example, consider the situation in which the second child has ancestral genotype $g_{k_{2}}$(*l*) = {*b*, *c*} at locus *l*, and he/she has already chosen mother *m*_1_. Then the possible outcomes are

$$\begin{array}{lll} {p^{\prime }}_{1} & \propto & p_{1}P(g_{f}=\{a,c\}|f\text{ transmits }a,\bar{\omega }),\\ {p^{\prime }}_{2} & \propto & p_{2}P(g_{m_{1}}=\{a,c\}|m_{1}\text{ transmits }a,\bar{\omega }),\\ {p^{\prime }}_{3} & \propto & p_{2}P(g_{f}=\{b,b\}|f\text{ transmits }b,\bar{\omega })\times P(g_{m_{1}}=\{a,c\}|m_{1}\text{ transmits }a,\bar{\omega }),\\ {p^{\prime }}_{4} & \propto & p_{2}P(g_{f}=\{b,c\}|f\text{ transmits }b,\bar{\omega })\times P(g_{m_{1}}=\{a,b\}|m_{1}\text{ transmits }a,\bar{\omega }). \end{array}$$

**Table 2:** 

$\phi_{k_{1}}$(*l*)	$\phi_{k_{2}}$(*l*)	${\bar{g}}_{f}$(*l*)	${\bar{g}}_{m_{1}}$(*l*)	P
0	1	{*a*, *c*}	{*a*, *b*}	${p^{\prime }}_{1}$
1	0	{*b*, ∅}	{*a*, *c*}	${p^{\prime }}_{2}$
1	0	{*b*, *b*}	{*a*, *c*}	${p^{\prime }}_{3}$
1	1	{*b*, *c*}	{*a*, *b*}	${p^{\prime }}_{4}$

Let the third child with ancestral genotype $g_{k_{3}}$(*l*) = {*b*, *d*} choose mother *m*_2_, who currently has ancestral genotype $g_{m_{2}}$(*l*) = {*d*, ∅}. Then the possible outcomes are

**Table 3:** 

$\phi_{k_{1}}$(*l*)	$\phi_{k_{2}}$(*l*)	$\phi_{k_{3}}$(*l*)	${\bar{g}}_{f}$(*l*)	${\bar{g}}_{m_{1}}$(*l*)	$g_{m_{2}}$(*l*)	P
1	0	0	{*b*, ∅}	{*a*, *c*}	{*d*, ∅}	${p^{\prime \prime }}_{1}$
1	0	0	{*b*, *b*}	{*a*, *c*}	{*d*, ∅}	${p^{\prime \prime }}_{2}$
1	0	0	{*b*, ∅}	{*a*, *c*}	{*d*, *d*}	${p^{\prime \prime }}_{3}$
1	0	0	{*b*, *b*}	{*a*, *c*}	{*d*, *d*}	${p^{\prime \prime }}_{4}$
1	0	1	{*b*, *d*}	{*a*, *c*}	{*d*, *b*}	${p^{\prime \prime }}_{5}$
1	1	0	{*b*, *c*}	{*a*, *b*}	{*d*, ∅}	${p^{\prime \prime }}_{6}$
1	1	0	{*b*, *c*}	{*a*, *b*}	{*d*, *d*}	${p^{\prime \prime }}_{7}$

$$\begin{array}{lll} {p^{\prime \prime }}_{1} & \propto & {p^{\prime }}_{2},\\ {p^{\prime \prime }}_{2} & \propto & {p^{\prime }}_{2}P(g_{f}=\{b,b\}|f\text{ transmits }b,\bar{\omega })+2{p^{\prime }}_{3},\\ {p^{\prime \prime }}_{3} & \propto & {p^{\prime }}_{2}P(g_{m_{2}}=\{d,d\}|m_{2}\text{ transmits }d,\bar{\omega }),\\ {p^{\prime \prime }}_{4} & \propto & ({p^{\prime }}_{2}P(g_{f}=\{b,b\}|f\text{ transmits }b,\bar{\omega })+2{p^{\prime }}_{3})+P(g_{m_{2}}=\{d,d\}|m_{2}\text{ transmits }d,\bar{\omega }),\\ {p^{\prime \prime }}_{5} & \propto & {p^{\prime }}_{2}P(g_{f}=\{b,d\}|f\text{ transmits }b,\bar{\omega })\times P(g_{m_{2}}=\{b,d\}|m_{2}\text{ transmits }d,\bar{\omega }),\\ {p^{\prime \prime }}_{6} & \propto & {p^{\prime }}_{4},\\ {p^{\prime \prime }}_{7} & \propto & {p^{\prime }}_{4}P(g_{m_{2}}=\{d,d\}|m_{2}\text{ transmits }d,\bar{\omega }). \end{array}$$

Note that now the event {$\phi_{k_{1}}$(*l*) = 0} has zero probability.

At the end of this sequential step children *k*_1_, ..., *k*_*n *_have chosen new mothers in a way which is compatible with their ancestral genotypes and the information about the genotypes of the parents carried by the modified configuration $\bar{\omega }$. We have also produced a joint sampling distribution for the phases of the children and the ancestral genotypes of the parents. Therefore we could complete the block-update by sampling, independently at each marker locus, the parental phases of the children's alleles and the ancestral genotypes of the parents from the joint sampling distribution above, and by transmitting recursively the new ancestral alleles upwards to the ancestors as in section A.6.2. However, this strategy would not take into account linkage between the marker loci, which is considered next.

#### A.7.2 Sampling the phases of the children's and parents' alleles jointly across the marker loci

We continue the block-update by computing for each parent *j *involved in the update and for each locus *l *the conditional probability of the parental phase *ϕ*_*j*_(*l*) given the transmitted genotype *g*_*j*_(*l*) and the genotypes of his/her ancestors under the modified configuration $\bar{\omega }$. Namely, as in equations (14–17),

$$\begin{array}{lll} P(\phi_{j}(l)=0|g_{j}(l)=\{a,b\},\bar{\omega }) & = & 1-P(\phi_{j}(l)=1|g_{j}(l)=\{a,b\},\bar{\omega })\\ & = & \{\begin{array}{ll} \frac{1}{2}, & \text{if }a=b=\emptyset ,\\ fr(a|f^{\prime },\bar{\omega })/[fr(a|f^{\prime },\bar{\omega })+fr(a|m^{\prime },\bar{\omega })], & \text{if }a\neq b=\emptyset ,\\ fr(a,b|f^{\prime },m^{\prime },\bar{\omega })/[fr(a,b|f^{\prime },m^{\prime },\bar{\omega })+fr(b,a|f^{\prime },m^{\prime },\bar{\omega })], & \text{if }a,b\neq \emptyset , \end{array} \end{array}$$

where *f' *and *m' *are the father and the mother of *j*.

We then combine, at every locus *l*, the product of the conditional phase distribution of the parents together with the joint distribution of the phases of the children and of the ancestral genotypes of the parents, as given in the previous section. By doing this, we obtain a joint distribution for the parental phases of the children and of the parents at locus *l*. In turn, the phases of the children together with the phases of the parents determine the grandparental origins of the alleles of the children. This gives a joint distribution for the grandparental origins of the children at locus *l*, which will be denoted by

$$Q_{l}(\psi_{2k_{1}-1}(l),\psi_{2k_{1}}(l),...,\psi_{2k_{n}-1}(l),\psi_{2k_{n}}(l))=Q_{l}(\phi .(l)).$$

We now include in the sampling distribution the likelihood contribution from the consecutive marker loci. Recall that the recombination likelihood from the children's haplotypes is given by

$$\prod_{l=1}^{L-1}R_{l,l+1}(\psi .(l),\psi .(l+1)),$$

where

$$R_{l,l+1}(\psi .(l),\psi .(l+1))=\rho {\left(l,l+1\right)}^{\#\{j\in J:\psi_{j}(l)\neq \psi_{j}(l+1)\}}{\left(1-\rho (l,l+1)\right)}^{\#\{j\in J:\psi_{j}(l)=\psi_{j}(l+1)\}}.$$

Here *ψ*_*j*_(*l*) ∈ {0, 1} is the grandparental origin of the haplotype *j *at locus *l*, *ρ*(*l*, *l *+ 1) is the recombination fraction between marker loci *l *and *l *+ 1, and *J *= {2*k*_1 _- 1, 2*k*_1_, ..., 2*k*_*n *_- 1, 2*k*_*n*_}. We use the forward-backward Baum-Viterbi algorithm to sample (and to compute the sampling distribution of) the grandparental origins (*ψ*_*j*_(*l*) : *j *∈ *J*, *l *= 1, ..., *L*) from the joint distribution proportional to

$$Q_{L}(\psi .(l))\prod_{l=1}^{L-1}\{Q_{l}(\psi .(l))R_{l,l+1}(\psi .(l),\psi .(l+1))\}.$$

Note that

$$R_{l,l+1}(\psi .(l),\psi .(l+1))=\prod_{j\in J}r_{l,l+1}(|\psi_{j}(l)-\psi_{j}(l+1)|),$$

where *r*_*l*, *l*+1_(*x*) = *ρ*(*l*, *l *+ 1)^*x*^(1 - *ρ*(*l*, *l *+ 1))^1-*x*^.

In the forward part of the algorithm, starting with $\tilde{Q}$_1 _(*ψ*.(*l*)) = *Q*_1_(*ψ*.(*l*)) we compute recursively for *l *= 1, ..., *L *- 1 the updated probability distributions

$${\tilde{Q}}_{l+1}(\psi .(l+1))\propto Q_{l+1}(\psi .(l+1))\left(\sum_{y.\in {\left\{0,1\right\}}^{J}}\left\{{\tilde{Q}}_{l}(y.\right)\prod_{j\in J}r_{l,l+1}(|\psi_{j}(l+1)-y_{j}|)\}\right)$$

On the right hand side appears a convolution in the commutative group ({0, 1}^2*n*^, +) which is computed efficiently by discrete Fourier transforms [31]. In the backward part of the algorithm we sample first *ψ*.(*L*) ~ $\tilde{Q}$_*L*_, and then iteratively *ψ*.(*l*) given *ψ*.(*l *+ 1) from the distribution

$$\begin{array}{cc} C_{l}\times {\tilde{Q}}_{l}(\psi .(l))\prod_{j\in J}r_{l,l+1}(|\psi_{j}(l+1)-\psi_{j}(l)|), & l=L-1,L-2,...,1, \end{array}$$

with normalizing constant *C*_*l *_= *Q*_*l*+1_(*ψ*.(*l *+ 1))/$\tilde{Q}$_*l*+1_(*ψ*.(*l *+ 1)).

Note that at any marker locus *l *different combinations of the children's phases and parents' phases may lead to the same vector *ψ*.(*l*) of children's grandparental origins. Therefore an additional step is required where, independently across the marker loci, the children's and parents' phases are sampled, conditionally on sampled grandparental origins of the children. This will determine also the ancestral genotypes transmitted to the parents. The block proposal is completed by transmitting the new ancestral alleles from the parents to their ancestors in the upper part of the ancestral graph. This is done exactly as in sections A.6.2 and A.6.3.

#### Remarks

(1) The computational complexity of this block update grows linearly with *L*, the number of markers, and exponentially with *n*, the number of children involved in the block update. In practice we restrict our sampling algorithm to values *n *≤ 7.

(2) In this block update we are sampling a full meiosis vector (*ψ*_*j*_(*l*) : *j *∈ *J*, *l *= 1, ..., *L*) which contains also the recombination pattern in the non-ancestral part of the haplotype. We have to proceed in this way, since it is not straightforward to sample the meiosis pattern only at the loci carrying ancestral alleles (the first-order Markov property across loci is lost). This means that we are temporarily extending the state space of the Markov chain algorithm with auxiliary variables. A theoretical justification is given in A.3. Note that, in order to compute the acceptance probability of this block update, we need to sample the meiosis pattern on the non-ancestral part of the haplotypes of the children as specified in the old configuration *ω*. This can be done directly by using the Kruglyak and Lander algorithm [31]. Once we have sampled the block update and computed the Hastings ratio, we erase the recombination pattern in the non-ancestral part of the haplotype and keep only the information about the paths of the ancestral alleles. 

(3) In the construction of this block proposal we have included the recombination likelihood from the haplotypes of the children but not the recombination likelihood from the haplotypes of the grandchildren and of the ancestors. Also these recombination likelihood terms contribute to the acceptance probability of the proposal.

### A.8 Block-update III: Sex-switching

We introduce one more update step into the algorithm. Consider the bipartite graph formed by the individuals in generation *t *> 0, where two nodes are connected by an edge if and only if the corresponding individuals have at least one common child. This bipartite graph is decomposable into connected components. We select a random connected component and obtain the proposal configuration $\bar{\omega }$ by switching the sexes of all the individuals in the selected component. Note that the prior distribution of the ancestral graph is not invariant under sex-switching (except for particular choices of the parameters *α *and *β*), but the distribution of the geneflow on the ancestral graph is, since our model for recombination does not depend on sex. Therefore, only the prior of the ancestral graph contributes to the Hastings' ratio which is given by min(1, *P*(*G*($\bar{\omega }$))/*P*(*G*(*ω*)). This simple update is important for the mixing of the sampler, since a fixed sex assignement would unnecessarily restrict the mating possibilities.

## B Sampling the parental phases conditionally on the partially observed haplotypes of the children: a Viterbi algorithm

Consider a parent whose (fully observed) genotype at locus *l *is $\tilde{g}(l)=\{{\tilde{g}}^{0}(l),{\tilde{g}}^{1}(l)\}\subseteq E_{l}$, where the two alleles are arbitrarily ordered. Let *ϕ *= (*ϕ*(1), ..., *ϕ*(*L*)) ∈ {0, 1}^*L *^be the phase vector of the parent, i.e. if allele ${\tilde{g}}^{0}(l)$ was inherited from the (grand)father, then *ϕ*(*l*) = 0, whereas if it came from the (grand)mother, then *ϕ*(*l*) = 1.

For each of the *n *children of the parent we introduce an origin vector ${\tilde{\zeta }}_{i}=({\tilde{\zeta }}_{i}(1),...,{\tilde{\zeta }}_{i}(L))\in {\left\{0,1\right\}}^{L}$, which together with the genotype of the parent determines the haplotype *h*_*i *_= (*h*_*i*_(1), ..., *h*_*i*_(*L*)) inherited by the *i*-th child from the parent as follows:

$$h_{i}(l)=\{\begin{array}{ll} {\tilde{g}}^{0}(l), & \text{if }{\tilde{\zeta }}_{i}(l)=0,\\ {\tilde{g}}^{1}(l), & \text{if }{\tilde{\zeta }}_{i}(l)=1. \end{array}$$

Note that the origin vector ${\tilde{\zeta }}_{i}$ together with the phase vector of the parent *ϕ *determines the grandparental origins and the recombination pattern for the haplotype *h*_*i*_.

Next we introduce a censoring mechanism *δ*_*i *_∈ {0, 1}^*L *^on haplotype *i *that is independent of *ϕ *and *h*_*i*_. We define the partially censored origin vector *ζ*_*i *_= (*ζ*_*i*_(1), ..., *ζ*_*i*_(*L*)) by setting

$$\zeta_{i}(l)=\{\begin{array}{ll} {\tilde{\zeta }}_{i}(l), & \text{if }\delta_{i}(l)=1\;(\text{uncensored allele}),\\ \emptyset , & \text{if }\delta_{i}(l)=0\;(\text{censored allele}). \end{array}$$

Given *ζ*_*i *_for all *i *= 1, ..., *n*, we define the partially censored parental genotype *g*(*l*) = {*g*^0^(*l*), *g*^1^(*l*)} ⊆ *E*_*l *_∪ {∅} as follows:

$$g^{j}(l)=\{\begin{array}{ll} {\tilde{g}}^{j}(l), & \text{if }\{i:1\leq i\leq n\text{ and }\zeta_{i}(l)=j\}\neq \emptyset ,\\ \emptyset , & \text{otherwise }({\tilde{g}}^{j}(l)\text{ is not ancestral}). \end{array}$$

Now the problem is to sample the parental phase vector *ϕ *conditionally on the children's partially censored origin vectors *ζ*_*i*_, *i *= 1, ..., *n*, the partially censored parent's genotype vector *g*, and the information available on the genotypes of the grandparents.

Without loss of generality we assume that *g*(*l*) ≠ {∅, ∅} for all *l*, since we can skip the loci where the genotype of the parent is completely censored. We assume that, *a priori*, the phases *ϕ*(*l*), *l *= 1, ..., *L*, are independent with respective distributions *π*(0, *l*) = (1 - *π*(1, *l*)) ∈ [0, 1]. The prior distributions *π*(*ϕ*(*l*), *l*) can be specified by using the information available on the genotypes of the grandparents as explained in section A.4.

Given the censoring pattern *δ*_*i *_on haplotype *i*, we denote the last uncensored locus up to *l *by *j*(*δ*_*i*_, *l*) := max{*k *≤ *l *: *δ*_*i*_(*k*) = 1} if such a locus exists, and otherwise set *j*(*δ*_*i*_, *l*) := 0. Let $\varepsilon_{l}:=\left\{j\left(\delta_{i},l\right):1\leq i\leq n\text{ and }j\left(\delta_{i},l\right)>0\right\}$ and *j*(*δ*_*i*_, *l*) > 0}. Note that ${ℰ}_{l}\subseteq ({ℰ}_{l-1}\cup \{l\})$ and since by assumption the parent transmits at least one allele to at least one child at each locus, *l *∈ ${ℰ}_{l}$ = {*l*} ⊆ {1, ..., *l*} for all *l*. Note also that without censoring, we would have simply ${ℰ}_{l}=\{l\}$ for all *l*.

We then consider the process ${\bar{\phi }}_{l}=(\phi (k):k\in {ℰ}_{l})\in {\left\{0,1\right\}}^{\left|{ℰ}_{l}\right|}$ for *l *= 1, ..., *L*. This is a Markov chain whose transition law $P({\bar{\phi }}_{l}|{\bar{\phi }}_{l-1})$ is simply described: the coordinates *ϕ*(*k*) do not change for $k\in ({ℰ}_{l-1}\cap {ℰ}_{l})$, whereas *ϕ*(*l*) is sampled independently from the parental phase prior *π*(*ϕ*(*l*); *l*). At each locus *l *> 1 we observe *ζ*(*l*) = (*ζ*_1_(*l*), ..., *ζ*_*n*_(*l*)) where only the components in

*K*(*l*) = {*i *: *ζ*_*i*_(*l*) ≠ ∅ and *j*(*δ*_*i*_, *l *- 1) > 0}

contribute to the likelihood of the phase vector (*ϕ*(1), ..., *ϕ*(*l*)) by a factor

$$\begin{array}{l} P(\zeta (l)|{\bar{\phi }}_{l},{\bar{\phi }}_{l-1},{ℋ}_{l-1})=\\ \prod_{i\in K(l)}R(1(\phi (l)=\phi (j(\delta_{i},l-1))),1(\zeta_{i}(l)=\zeta_{i}(j(\delta_{i},l-1))),\rho (j(\delta_{i},l-1),l)). \end{array}$$

Here *R*(0, 0, *ρ*) = *R*(1, 1, *ρ*) = 1 - *ρ*, *R*(1, 0, *ρ*) = *R*(0, 1, *ρ*) = *ρ*, *ρ *(*k*, *l*) is the recombination fraction between the loci *k *and *l *and ${ℋ}_{l}$ := {*ζ*(*k*) : *k *≤ *l*}.

Summarizing: If we do not have censoring, (*ϕ*(*l*), *ζ*(*l*)) is a Markov chain with hidden state *ϕ*(*l*) ∈ {0, 1}, and given the censoring pattern (*δ*_*i *_: *i *= 1, ..., *n*), we have constructed a Markov process ${\bar{\phi }}_{l}$ with an enlarged state space ${ℰ}_{l}$ depending on the locus *l*, such that the conditional distribution of the observation process *ζ*(*l*), given ${\bar{\phi }}_{l}$ and (*ϕ*(*k*), *ζ*(*k*) : *k *<*l*), depends only on ${\bar{\phi }}_{l}$, ${\bar{\phi }}_{l-1}$ and the previous observations (*ζ*(*j*(*δ*_*i*_, *l *- 1)) : *i *≤ *n*) at the last uncensored loci, i.e. given the censoring pattern *δ*_*i*_, (${\bar{\phi }}_{l}$, (*ζ*_*i*_(*j*(*δ*_*i*_, *l*)) : *i *≤ *n*)) is a Markov process.

After having enlarged the state space, we are back to the framework of hidden Markov models, and we can use the Viterbi algorithm to sample from the joint posterior distribution the vector $\left({\bar{\phi }}_{1},...,{\bar{\phi }}_{L}\right)$ (which contains the same information as the vector (*ϕ*(1), ..., *ϕ*(*L*)), conditionally on the observations (*ζ*_*i*_(1), ..., *ζ*_*i*_(*L*) : *i *= 1, ..., *n*). Next we sketch the algorithm.

At the first locus we have always ${\bar{\phi }}_{1}$ = (*ϕ*(1)) and $P({\bar{\phi }}_{1}|{ℋ}_{1})$ = *π*(*ϕ*(1); 1). For the following loci we compute sequentially the distribution of the phase vector ${\bar{\phi }}_{l}=(\phi (k):k\in {ℰ}_{l})\in {\left\{0,1\right\}}^{\left|{ℰ}_{l}\right|}$ conditionally on the data up to locus *l*:

$$P({\bar{\phi }}_{l}|{ℋ}_{l})=\sum_{{\bar{\phi }}_{l-1}\in {\left\{0,1\right\}}^{\left|{ℰ}_{l-1}\right|}}P({\bar{\phi }}_{l-1}|{ℋ}_{l-1})P({\bar{\phi }}_{l}|{\bar{\phi }}_{l-1},{ℋ}_{l}),$$

where

$$P({\bar{\phi }}_{l}|{\bar{\phi }}_{l-1},{ℋ}_{l})\propto P({\bar{\phi }}_{l}|{\bar{\phi }}_{l-1})P(\zeta (l)|{\bar{\phi }}_{l},{\bar{\phi }}_{l-1},{ℋ}_{l-1}).$$

Next, we sample ${\bar{\phi }}_{L}=(\phi (k):k\in {ℰ}_{L})$ from $P({\bar{\phi }}_{L}|{ℋ}_{L})$, and continue sequentially backwards from locus *L *to locus 1. Having sampled ${\bar{\phi }}_{L},...,{\bar{\phi }}_{l+1}$, we sample ${\bar{\phi }}_{l}$ from the conditional distribution

$$P({\bar{\phi }}_{l}|{\bar{\phi }}_{l+1},{ℋ}_{l+1})\propto P({\bar{\phi }}_{l}|{ℋ}_{l})P({\bar{\phi }}_{l+1}|{\bar{\phi }}_{l},{ℋ}_{l+1})$$

By the same method one computes the posterior probability of a given sample (*ϕ*(1), ..., *ϕ*(*L*)), and it would also be possible to find the phase vector of maximal posterior probability by using dynamic programming.

In a similar fashion, we can compute recursively the marginal distribution of the data. For the given sampling pattern, at the first locus we have simply

$$P({\bar{\phi }}_{1},\zeta (1))=\pi (\phi (1);1)P(\zeta (1)),\text{ with }P(\zeta (1))=2^{-(\#\{i:\delta_{i}(1)=1\})}.$$

Then we compute recursively, for 1 <*l *≤ *L*,

$$\begin{array}{l} P(\zeta (1),\zeta (2),...,\zeta (l),{\bar{\phi }}_{l})=\sum_{{\bar{\phi }}_{l-1}\in {\left\{0,1\right\}}^{\left|{ℰ}_{l-1}\right|}}\{P(\zeta (1),...,\zeta (l-1),{\bar{\phi }}_{l-1})\times \\ P({\bar{\phi }}_{l}|{\bar{\phi }}_{l-1})P(\zeta (l)|\zeta (l-1),{\bar{\phi }}_{l-1},{\bar{\phi }}_{l}\}, \end{array}$$

and finally

$$P(\zeta (1),\zeta (2),...,\zeta (L))=\sum_{{\bar{\phi }}_{L}\in {\left\{0,1\right\}}^{\left|{ℰ}_{L}\right|}}P(\zeta (1),...,\zeta (l),{\bar{\phi }}_{L}).$$

It is quite remarkable that this algorithm does not need to sample the missing data. The possiblity of sampling the parental phases, by conditioning on the haplotypes of the children, is crucial for the mixing of the MCMC algorithm. Sampling the phases from the prior only is unlikely to produce a reasonable recombination pattern on the haplotypes of the children, with the consequence that the acceptance probability of the whole move will be very low.

## References

[B1] Gao G, Hoeschele I, Sorensen P, Du FX (2004). Conditional probability methods for haplotyping in pedigrees. Genetics.

[B2] Lin S, Cutler DJ, Zwick ME, Chakravarti A (2002). Haplotype inference in random population samples. Am J Hum Genet.

[B3] Blouin MS (2003). DNA-based methods for pedigree reconstruction and kinship analysis in natural populations. Trends Ecol Evol.

[B4] Cowell RG, Mostad P (2003). A clustering algorithm using DNA marker information for sub-pedigree reconstruction. J Forensic Sci.

[B5] Lange EM, Lange K (2004). Powerful allele sharing statistics for nonparametric linkage analysis. Hum Hered.

[B6] Du FX, Hoeschele I (2000). A note on algorithms for genotype and allele elimination in complex pedigrees with incomplete genotype data. Genetics.

[B7] Pirinen M, Gasbarra D (2006). Finding consistent gene transmission patterns on large and complex pedigrees. IEEE/ACM TCBB.

[B8] Falush D, Stephens M, Pritchard J (2003). Inference of population structure using multilocus genotype data: linked loci and correlated allele frequencies. Genetics.

[B9] Corander J, Waldmann P, Sillanpää MJ (2003). Bayesian analysis of genetic differentiation between populations. Genetics.

[B10] Gasbarra D, Pirinen M, Sillanpää MJ, Salmela E, Arjas E (2007). Estimating genealogies from unlinked marker data: a Bayesian approach. Theor Pop Biol.

[B11] Jannink JL, Wu XL (2003). Estimating allelic number and identity in state of QTLs in interconnected families. Genet Res.

[B12] Pérez-Enciso M (2003). Fine mapping of complex trait genes combining pedigree and linkage disequilibrium information: a Bayesian unified framework. Genetics.

[B13] Meuwissen THE, Goddard ME (2004). Mapping multiple QTL using linkage disequilibrium and linkage analysis information and multitrait data. Genet Sel Evol.

[B14] Sobel E, Papp JC, Lange K (2002). Detection and integration of genotyping errors in statistical genetics. Am J Hum Genet.

[B15] Sobel E, Sengul H, Weeks DE (2001). Multipoint estimation of identity-by-descent probabilities at arbitrary positions among marker loci on general pedigrees. Hum Hered.

[B16] Thompson EA, Heath SC, Seillier-Moseiwitch F (1999). Estimation of conditional multilocus gene identity among relatives. Statistics in Molecular Biology and Genetics: selected proceedings of a 1997 joint AMS-IMS-SIAM summer conference on statistics in molecular biology, IMS Lecture Note-Monograph Series.

[B17] Mao Y, Xu S (2005). A Monte Carlo algorithm for computing IBD matrices using incomplete marker information. Heredity.

[B18] Hernández-Sánchez J, Haley CS, Wooliams JA (2006). Prediction of IBD based on population history for fine gene mapping. Genet Sel Evol.

[B19] Kingman JFC (1982). The coalescent. Stochastic Proc Appl.

[B20] Hudson RR (1983). Properties of a neutral allele model with intragenic recombination. Theor Pop Biol.

[B21] Griffths RC, Marjoram P (1996). Ancestral inference from samples of DNA sequences with recombination. J Comp Biol.

[B22] Stephens M, Smith NJ, Donnelly P (2001). A new statistical method for haplotype reconstruction from population data. Am J Hum Genet.

[B23] Larribe F, Lessard S, Schork NJ (2002). Gene mapping via the ancestral recombination graph. Theor Pop Biol.

[B24] Morris AP, Whittaker JC, Balding DJ (2002). Fine-scale mapping of disease loci via shattered coalescent modeling of genealogies. Am J Hum Genet.

[B25] Zöllner S, Pritchard JK (2005). Coalescent-based association mapping and fine mapping of complex trait loci. Genetics.

[B26] Kuhner M, Felsenstein J (2000). Sampling among haplotype resolutions in a coalescent-based genealogy sampler. Genet Epidemiol.

[B27] Fearnhead P, Donnelly P (2001). Estimating recombination rates from population genetic data. Genetics.

[B28] Beaumont MA, Zhang W, Balding DJ (2002). Approximate Bayesian computation in population genetics. Genetics.

[B29] Marjoram P, Molitor J, Plagnov V, Tavaré S (2003). Markov chain Monte Carlo without likelihoods. Proc Natl Acad Sci USA.

[B30] Gasbarra D, Sillanpää MJ, Arjas E (2005). Backward simulation of ancestors of sampled individuals. Theor Pop Biol.

[B31] Kruglyak L, Lander ES (1998). Faster multipoint linkage analysis using Fourier transforms. J Comput Biol.

[B32] Stephens M, Scheet P (2005). Accounting for decay of linkage disequilibrium in haplotype inference and missing-data imputation. Am J Hum Genet.

[B33] Lynch M (1988). Estimation of relatedness by DNA fingerprinting. Mol Biol Evol.

[B34] Li CC, Weeks DE, Chakravarti A (1993). Similarity of DNA fingerprints due to chance and relatedness. Hum Hered.

[B35] Lynch M, Ritland K (1999). Estimation of pairwise relatedness with molecular markers. Genetics.

[B36] Wang J (2002). An estimator for pairwise relatedness using molecular markers. Genetics.

[B37] Rousset F (2002). Inbreeding and relatedness coefficients: what do they measure?. Heredity.

[B38] van der Meulen M, te Meerman GJ, Pawlowitzki IH, Edwards JH, Thompson EA (1997). Association and haplotype sharing due to identity by descent, with an application to genetic mapping. Genetic Mapping of Disease Genes.

[B39] te Meerman GJ, van der Meulen MA (1997). Genomic sharing surrounding alleles identical by descent: effects of genetic drift and population growth. Genet Epidemiol.

[B40] Beckmann L, Thomas DC, Fischer C, Chang-Claude J (2005). Haplotype sharing analysis using Mantel statistics. Hum Hered.

[B41] Hein J, Schierup MH, Wiuf C (2005). Gene Genealogies, Variation and Evolution: A Primer in Coalescent Theory.

[B42] Heath SC (1997). Markov chain Monte Carlo methods for radiation hybrid mapping. J Comp Biol.

[B43] Del Moral P (2004). Feynman-Kac Formulae: Genealogical and Interacting Particle Systems with Applications.

[B44] Lund MS, Sorensen P, Guldbrandtsen B, Sorensen DA (2003). Multitrait fine mapping of quantitative trait loci using combined linkage disequilibria and linkage analysis. Genetics.

[B45] Yi N, Xu S (2000). Bayesian mapping of quantitative trait loci under the identity-by-descent-based variance component model. Genetics.

[B46] Heath SC (1997). Markov chain Monte Carlo segregation and linkage analysis for oligogenic models. Am J Hum Genet.

[B47] Sillanpää MJ, Arjas E (1998). Bayesian mapping of multiple quantitative trait loci from incomplete inbred line cross data. Genetics.

[B48] Uimari P, Sillanpää MJ (2001). Bayesian oligogenic analysis of quantitative and qualitative traits in general pedigrees. Genet Epidemiol.

[B49] Robert CP, Casella G (1999). Monte Carlo Statistical Methods.

[B50] Besag J, Green E, Higdon D, Mengersen KL (1995). Bayesian computation and stochastic systems. Stat Sci.

